# Wildlife resistance and protection in a changing New England landscape

**DOI:** 10.1371/journal.pone.0239525

**Published:** 2020-09-24

**Authors:** Schuyler B. Pearman-Gillman, Matthew J. Duveneck, James D. Murdoch, Therese M. Donovan

**Affiliations:** 1 Vermont Cooperative Fish and Wildlife Research Unit, Burlington, Vermont, United States of America; 2 Rubenstein School of Environment and Natural Resources, University of Vermont, Burlington, Vermont, United States of America; 3 Harvard Forest, Harvard University, Petersham, Massachusetts, United States of America; 4 Wildlife and Fisheries Biology Program, Rubenstein School of Environment and Natural Resources, University of Vermont, Burlington, Vermont, United States of America; 5 United States Geological Survey, Burlington, Vermont, United States of America; Cornell University, UNITED STATES

## Abstract

Rapid changes in climate and land use threaten the persistence of wildlife species. Understanding where species are likely to occur now and in the future can help identify areas that are resistant to change over time and guide conservation planning. We estimated changes in species distribution patterns and spatial resistance in five future scenarios for the New England region of the northeastern United States. We present scenario-specific distribution change maps for nine harvested wildlife species, identifying regions of increasing, decreasing, or stable habitat suitability within each scenario. Next, we isolated areas where species occurrence probability is high (p > 0.7) and resistant to change across all future scenarios. Resistance was also evaluated relative to current land protection to identify patterns in and out of Protected Areas (PAs). Generally, species distributions declined in area over the 50-year assessment period (2010–2060), with the greatest average declines occurring for moose (-40.9%) and wild turkey (-22.1%). Species resistance varied considerably across the region, with coyote demonstrating the highest average regional resistance (91.81% of the region) and moose demonstrating the lowest (0.76% of the region). At the state level, average focal species resistance was highest in Maine (the largest state) and lowest in Massachusetts. Many of the focal species showed high overlap in resistance and land protection. Coyote, white-tailed deer, and black bear had the highest probability of resistance, given protection, while moose and wild turkey had the highest probability of protection, given resistance. Overall, relatively small portions of New England—ranging between 0.25% and 21.12%–were both protected and resistant for the focal species. Our results provide estimates of resistance that can inform conservation planning for commonly harvested species that are important ecologically, economically, and culturally to the region. Expanding protected area coverage to include resistant areas may provide longer term benefits to these species.

## Introduction

Resilience describes a system’s broad ability to cope with disturbances without changing state [1]. Spatial resilience further describes a system or landscape capacity to support ecosystems and biodiversity over space and time in response to disturbance [2–4]. Resilience studies often focus on broad concepts, such as conserving biodiversity and ecosystem function, or on specific taxa of interest (e.g., avian species), or groups of vulnerable species (e.g., endangered or climate-sensitive species) [2,5–8]. For example, Anderson et al. [9] evaluated resilience based on the ability of a geophysical setting to sustain a diversity of species, natural communities, and ecological relationships. This approach targeted the broader preservation of biodiversity and identified sites throughout eastern North America that are likely to consistently support plants and animals over the long term despite changes to climate and landscape conditions.

Because ecosystem resilience is complex and challenging to quantify, evaluating different aspects of resilience can provide important insights and perspectives for conservation. Resistance is an inherent aspect of resilience that identifies which systems, species, or locations are least vulnerable to change in the face of disturbance [1,4,10,11]. Some studies suggest that resilience depends on the capacity of a species or ecosystem to resist change as well as the spatial and environmental context in which that system or species exists [12–16]. Thus, using spatial approaches to evaluate resistance can provide context for understanding resilience for conservation purposes.

**Fig 1:**
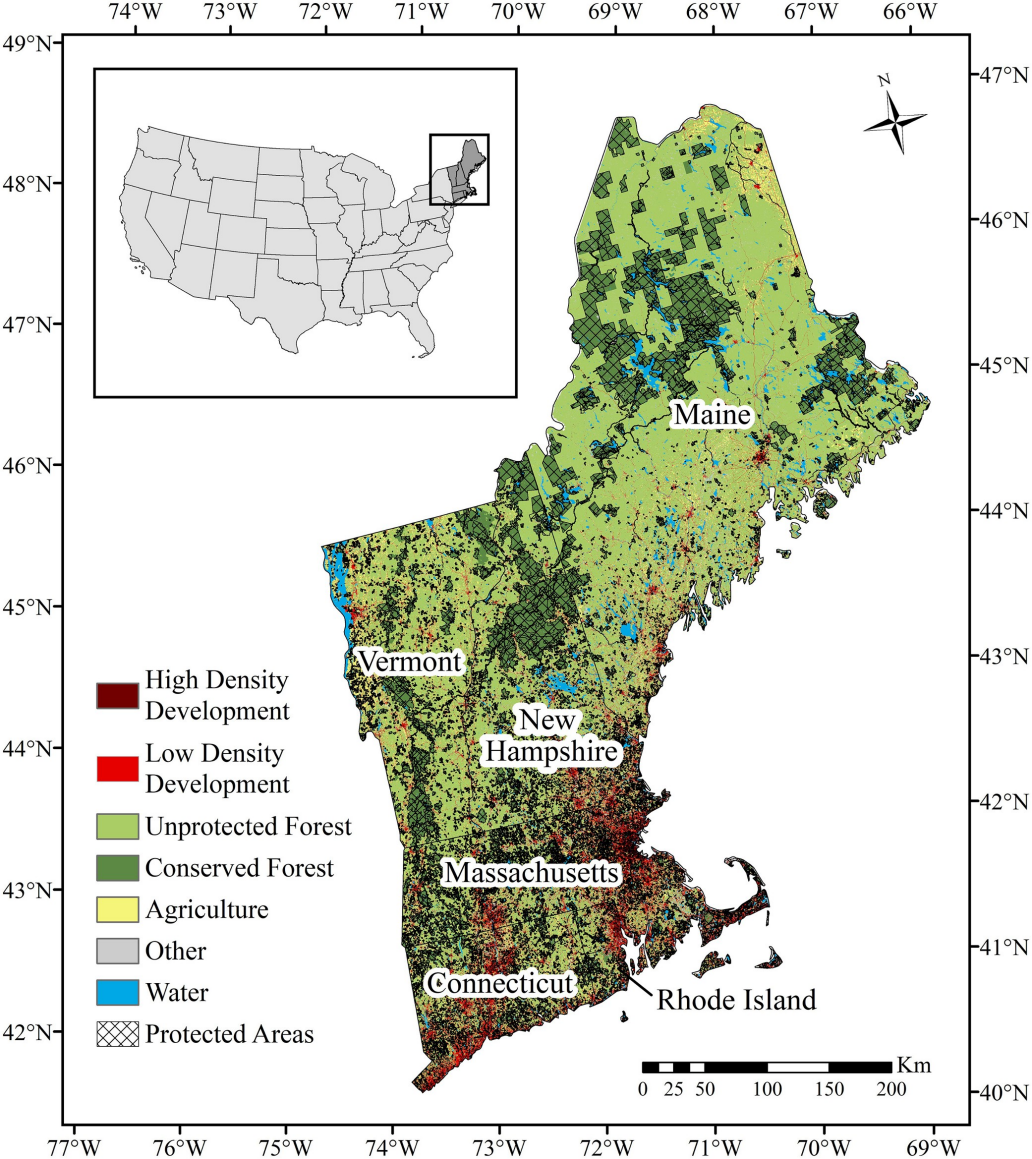
Map of the New England region where wildlife species resistance to change was studied. The New England study region encompasses six states—Connecticut, Maine, Massachusetts, New Hampshire, Rhode Island, and Vermont—and over 57,000 protected area parcels [28]. Data sources: U.S. Census Bureau [29], U.S. Geological Survey [28], and Plisinski et al. [30].

The New England region in the northeastern United States (186,458 km^2^; Fig 1) covers six states and has a long history of social, economic, and ecological change [17–19]. With the escalating pressures of population expansion, changing land use and development, climate change, and altered disturbance regimes, New England will be subject to rapid modification over the next half-century [17,20–23]. These environmental changes can significantly alter the quality, availability, and connectivity of natural systems, and subsequently influence the distribution of wildlife species [24–26]. The impacts of change on harvested wildlife species are of particular interest in New England because of their ecological, economic, and cultural importance [27].

Effective long-term conservation and management of wildlife species requires a comprehensive understanding of species’ potential responses not only to environmental stressors and disturbances, but also to future policy and management actions [4]. Scenario-planning provides a powerful way to explore and understand hypothetical futures while explicitly acknowledging their inherent uncertainty [31,32]. By exploring possible futures, scenario-planning can help address uncertainties around socio-economic drivers and spatial dynamics of environmental change, and generate new insights about the complex, dynamic systems that impact wildlife futures [32,33].

**Fig 2:**
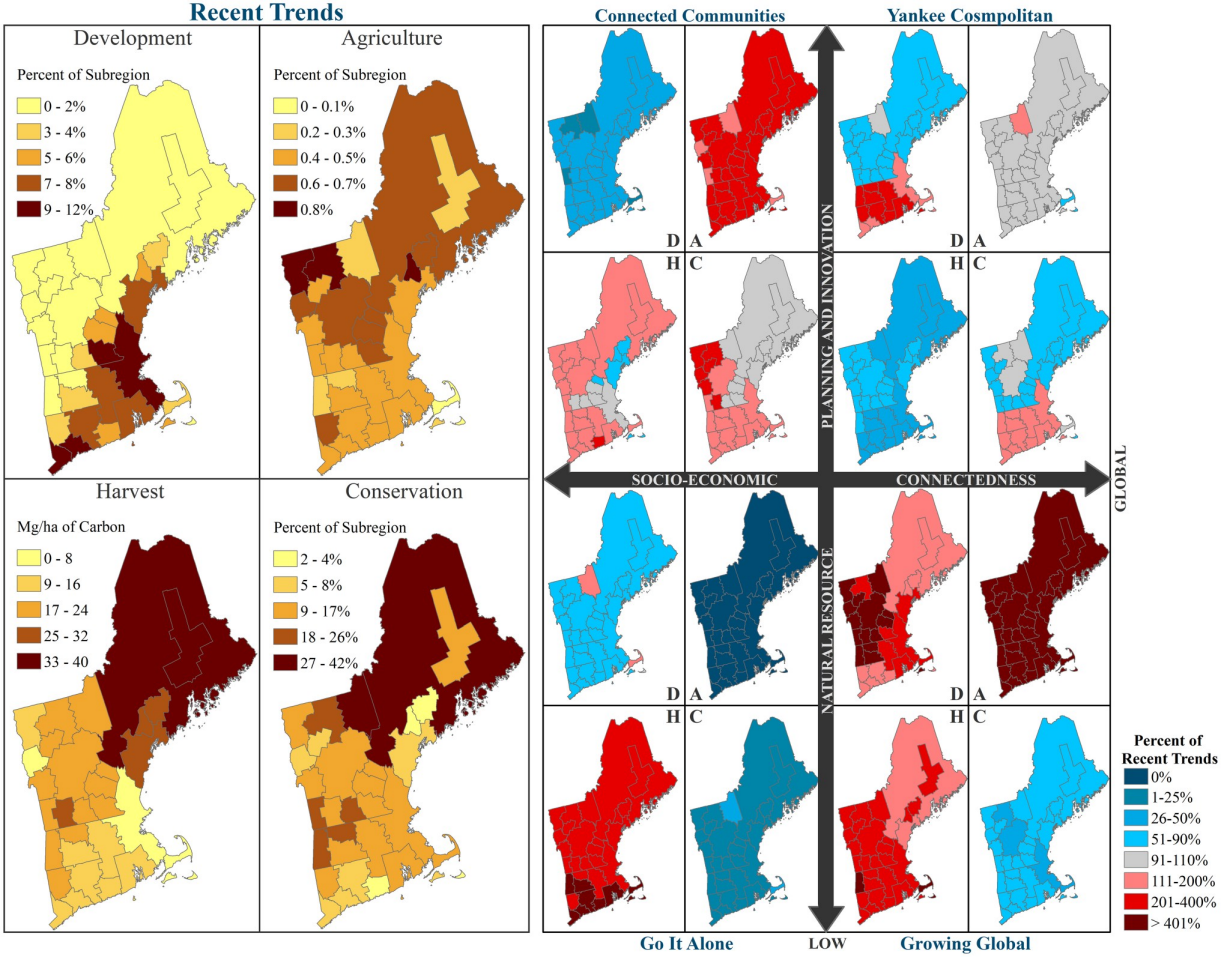
The NELFP scenarios used to estimate wildlife species future probability of occurrence throughout the New England region. Scenarios were built around two drivers of landscape change: 1) Natural Resource Planning & Innovation and 2) Socio-Economic Connectedness. The drivers form four alternatives scenarios to recent trends: “Connected Communities”, “Yankee Cosmopolitan”, “Go It Alone”, and “Growing Global”. Scenario-specific changes in development, agriculture, forest harvest, and conservation were simulated for the New England region over a fifty-year time period (2010 to 2060). Recent Trends scenario (left) displays the annual quantity of land cover and land use change broken down by subregion. The alternative NELFP scenarios (right) display the percent change from recent trends. Figure modified from a previously published figure in Thompson et al. [35]. Data sources: U.S. Census Bureau [29,36] and Plisinski et al. [30].

The New England Landscape Futures Project (NELFP), led by the Harvard Forest Long-Term Ecological Research program and the Scenarios, Services, and Society Research Coordination Network developed five plausible scenarios for how New England’s landscape may change over fifty-years (2010 to 2060). The NELFP simulations include a recent trends scenario (i.e., “Business-As-Usual”) and four alternative scenarios that were built around two drivers of social and ecological change (Fig 2): 1) Natural Resource Planning & Innovation (NRPI)–the extent to which the government and private sector invest in proactive land-use planning, ecosystem services, and technological advances for resource use—and 2) Socio-Economic Connectedness (SEC)–the local or global connectivity of population migration, economic markets, and climate policy [31]. These scenarios provide spatial projections of climate, forest structure and composition, development, and agriculture, making them well suited for spatially explicit assessments of wildlife futures. A previous study by Pearman-Gillman et al. [34] evaluated future distributions of harvested species under the NELFP scenarios and found that predicted distribution patterns varied considerably among the scenarios. However, all scenarios projected a decline in the spatial distribution for most species. The results highlighted uncertainty around species’ futures in the New England region and raised questions about species vulnerability and resistance to future change [34].

The NELFP scenarios capture a wide range of possible future conditions and provide an opportunity to spatially quantify resistance across New England for harvested wildlife species. With current distribution patterns serving as a baseline, predicted changes in species occurrence patterns can be evaluated *across* scenarios to identify areas where occurrence remains high and is resistant to future change on a species-by-species basis. Such analyses permit an evaluation of how well resistance aligns with the current conservation network. In New England, over 57,000 parcels—covering ~22% of the region’s land area—are currently under a conserved land status (Fig 1) [28]. These protected areas (PAs) are geographically defined parcels created in most cases to conserve habitat, species diversity, natural resources, and recreational values [37,38]. However, PAs are often located in high elevation and other undevelopable areas, and consequently may not align with areas that are resistant to future change [39]. Because protected areas are often treated as static entities that remain in the same place over time [37], it is essential to understand how existing land protection aligns with species future distributions and whether current reserve networks will support conservation targets in the future [40].

In the current era of rapid change, strategic land protection and proactive conservation planning will be critical for conserving natural landscapes [41]. Decision-makers frequently prioritize conservation on rare, threatened and endangered species [42], especially in the New England region. While it is crucial to protect rare and vulnerable species, it is also important to consider common species in conservation assessments and decisions. In New England, harvested wildlife species represent a group of ecologically, economically, and culturally important species that commonly occur throughout the region. Although many harvested species are of low conservation concern, these species directly influence other taxa, ecological functions, and management decisions [43–45]. Understanding how future change will impact harvested wildlife species can provide important context and support broader conservation of biological diversity and ecological functions, despite inevitable shifts in climate and land use [9,46].

We present a novel approach for assessing species spatial resistance to change using a scenario-based framework. We focus on nine ecologically and socio-economically relevant wildlife species and build a comprehensive understanding of how multiple landscape futures (the NEFLP scenarios) are likely to impact species occurrence across a large regional extent. We apply a systematic approach to 1) Map distribution change for each species under five alternative scenarios, 2) Identify areas on the landscape where species persist under individual scenarios and remain resistant to change across all scenarios, and 3) Evaluate resistance within protected areas and throughout the New England region.

## Methods

### Study area

The study area spanned 186,458 km^2^ in the northeastern United States and encompassed the six New England states: Connecticut, Rhode Island, Massachusetts, Vermont, New Hampshire, and Maine (Fig 1). The region is characterized by diverse topography [47,48], forest types [49,50], and land uses [23,51]. Climatic conditions vary greatly across the region, from humid subtropical climate in the southern coastal regions to subarctic conditions in the northern mountains [52–54]. With two-thirds of the region’s growing human population (14,853,290) concentrated in major metropolitan areas [55], New England is one of the most densely populated and forested regions in the United States. In 2010 –the start of the NELFP scenario timeline—approximately 80% of the region was covered by forest [23,51], with development (7.3% low density and 1.3% high density), agriculture (6.4%) and water (4.6%) comprising the majority of the non-forested landscape [23,56].

### Focal species

We focused our analysis on nine harvested wildlife species that occur widely throughout the New England region. This group included six carnivorans—American black bear (*Ursus americanus*), bobcat (*Lynx rufus*), coyote (*Canis latrans*), raccoon (*Procyon lotor*), red fox (*Vulpes vulpes*), and striped skunk (*Mephitis mephitis*); two ungulates—moose (*Alces alces*) and white-tailed deer (*Odocoileus virginianus*); and one Galliform—wild turkey (*Meleagris gallopavo*). Although largely associated with forests, these species have diverse habitat and home range requirements, varied sensitivity to human influences, and unique natural histories in the New England region [57].

### Objective 1—Map species distribution change

#### Scenario simulations

We used NELFP scenarios to estimate distribution change, persistence, and resistance for the focal species [31,58]. The NELFP scenarios included: “Connected Communities” (based on high NRPI and local SEC), “Yankee Cosmopolitan” (high NRPI and global SEC), “Go It Alone” (low NRPI and local SEC), and “Growing Global” (low NRPI and global SEC; Fig 2). A “Recent Trends” scenario was also included to provide a baseline projection based on recent trends in climate and land use change. This scenario represents a linear continuation of the land use and land cover changes observed between 1990 and 2010 (as defined by Thompson et al. [20]).

Each NELFP scenario followed a different trajectory of land cover and land-use change derived from the scenarios unique narrative (see [31,58] for detailed scenario narratives). Climate changes for each scenario stayed consistent based on the assumptions of the Representative Concentration Pathway (RCP) 8.5 emission scenario [22,59]. The scenario narratives were translated into spatial patterns of change using methods described by [20] and [22]. Briefly, these simulations were developed in two stages: first using a spatially explicit cellular land change model, Dinamica Environment for Geoprocessing Objects [60] and second using a forest landscape succession model, LANDIS-II [61].

We used maps of species probability of occurrence under recent conditions (2010) [62] and scenario simulated occurrence maps for the year 2060 [34] to evaluate changes in species occurrence probability under alternative future conditions. These maps were based on species distribution models (SDMs) developed by Pearman-Gillman et al. [62]. Models were developed from expert opinion data and evaluated the effects of combinations of 74 variables on occurrence probability. For each of the 5 scenarios, we compared the scenario-derived distribution maps against recent conditions distribution maps to assess potential changes (i.e., differences in species occurrence probabilities throughout New England). Current map cells were subtracted from superimposed projected map cells to calculate absolute change. Map cells with negative values represented locations of declining occurrence probability and cells with positive values represented locations of increasing occurrence probability. All maps were developed using the raster package [63] in the R statistical computing language [64].

### Objective 2—Identify areas of persistence and resistance

#### Persistence

For each species in each scenario, we identified areas of persistence in occurrence probabilities between 2010 and 2060. Species occurrence probabilities were evaluated on a cell-by-cell basis to develop binary persistence maps for each scenario. Assuming a conservation goal of maximizing occupancy of the harvested species across the landscape, we identified scenario-specific persistence using two criteria: 1) high occurrence probability (*p* > 0.70) under recent conditions (2010), and 2) high occurrence probability (*p* > 0.70) under scenario projected conditions (2060). Map cells (30 x 30 m) with high occurrence probabilities under both recent and scenario projected conditions were classified as persistent and coded 1; cells that failed to meet the persistence criteria were coded 0.

#### Resistance

We developed resistance maps for each species by identifying common areas of persistence *across* all five alternative scenarios. Resistance was determined by multiplying across the five scenario-specific binary persistence layers; map cells that met the resistance criteria under all five future scenarios were considered resistant and retained the value 1, while cells that failed to meet the criteria under one or more of the scenarios were converted to 0. Resistance statistics were calculated for each species and were compared across the focal group to indicate trends in species resistance to change.

Within resistant areas, changes in occurrence probability were additionally categorized into one of four resistance classes. Resistant map cells with relatively constant occurrence probabilities (i.e., change in occurrence probability < ±0.1) between 2010 and each of the five scenarios (2060) were classified as “resistant-constant”. Cells that consistently experienced an increase in occurrence probability (i.e., a positive change in occurrence probability of > 0.1) between 2010 and 2060 under all five scenarios were classified as “resistant-increasing”. Similarly, cells that consistently experienced a decrease in occurrence probability (i.e., a negative change in occurrence probability of > 0.1) were classified as “resistant-decreasing”. Cells that experienced inconsistent trends of change among the five scenarios (e.g., increased occurrence probability under one scenario and decreased occurrence probability under another scenario) were classified as “resistant-variable”. Summary statistics were calculated for each resistance class to provide regional information about patterns of change within resistant areas.

### Objective 3—Evaluate resistance within protected areas

We used species resistance maps and information from the National Inventory of Protected Areas [28] to evaluate the overlap between the current protected area network and each species resistance map. This inventory included protected lands from most federal land management agencies (e.g., the National Park Service, U.S. Forest Service, U.S. Fish and Wildlife Service), and integrates non-profit databases (e.g. The Nature Conservancy Fee Lands and Secured Lands aggregation, The Trust for Public Land Conservation Almanac, Ducks Unlimited Conservation and Recreation Lands) and the latest easement data from the National Conservation Easement Database.

We superimposed polygons from the Protected Areas Database of the U.S. (PAD-US version 2.0) [28] with species resistance layers and calculated zonal statistics for each Protected Area polygon in the New England region. We evaluated patterns of resistance in and out of the protected network and identified the PAs with the greatest resistance for individual species. Resistance scores were also calculated for each protected parcel based on mean resistance across all focal species. All statistics were calculated using R [64] and ArcGIS 10 Geographic Information System [65].

## Results

### Objective 1—Distribution change

American black bear, moose, red fox, and wild turkey were projected to have the largest change in occurrence probabilities throughout New England (Table 1). For example, black bear had an average occurrence probability (across all cells on the landscape) of 0.80 in the baseline projection at year 2010; under the Recent Trends scenario, the average occurrence probability decreased to 0.68 by year 2060 (a -15.3% change; Table 1). On average, all but one species (red fox) was projected to decline in distribution. For black bear, moose, and wild turkey, large localized shifts in occurrence probabilities led to moderate-to-large declines in average regional occurrence (-15.3%, -40.9, and -22.1, respectively; see S1 Fig for species distribution change maps). For red fox, moderate shifts in occurrence probabilities throughout New England led to relatively large increases (29.9%) in average regional occurrence (Table 1; S1 Fig). Scenario-specific changes in occurrence were relatively low for bobcat, coyote, raccoon, striped skunk, and white-tailed deer. For example, coyote occurrence was projected to decrease slightly (< -3.5%) in all 5 future scenarios, while white-tailed deer occurrence was projected to decrease slightly in some scenarios (e.g., Growing Global = -4.1%) and increase slightly in others (e.g., Recent Trends = +0.5%). For these species, localized increases and decreases in occurrence probability largely balanced out across the region, resulting in minimal change in average regional occurrence (Table 1; S1 Fig).

**Table 1:** Distribution change statistics for nine wildlife species in the New England region of the northeastern United States.

Species	Mean occurrence probability (2010)	Distribution change (%) in NELFP scenarios by year 2060					
		Recent Trends	Growing Global	Go It Alone	Yankee Cosmopolitan	Community Connectedness	Average
American black bear	0.80	-15.3	-19.0	-11.4	-17.0	-13.9	-15.3
Bobcat	0.67	-5.6	-5.6	-5.3	-7.1	-2.9	-5.3
Coyote	0.92	-3.1	-2.2	-3.1	-3.5	-2.6	-2.9
Moose	0.52	-51.8	-28.1	-19.5	-62.4	-42.8	-40.9
Raccoon	0.87	-5.6	-2.7	-6.0	-5.7	-5.6	-5.1
Red fox	0.64	29.8	30.4	29.6	29.7	30.0	29.9
Striped skunk	0.75	-6.0	-1.2	-6.3	-6.4	-5.3	-5.0
White-tailed deer	0.89	0.5	-4.1	-2.2	0.2	-0.7	-1.3
Wild turkey	0.68	-24.0	-16.7	-22.3	-24.2	-23.2	-22.1
Average		-10.5	-4.3	-7.2	-12.3	-8.6	

Mean occurrence probabilities were based on recent (2010) conditions and provide baseline distribution information for the region. Distribution change indicates the percent increase or decrease in regional occurrence probability between species 2010 distribution and each of the NELFP scenario simulated 2060 distributions. For example, black bear average occurrence probability under the recent trends projection (*p* = 0.68) represented a 15.3% decline in distribution from the recent conditions baseline (*p* = 0.80). See S1 Table for additional distribution change statistics.

### Objective 2—Persistence and resistance

#### Persistence

Scenario-specific areas of persistence (i.e., map cells with > 0.7 occurrence probabilities in 2010 *and* 2060) ranged between 0.99% of the landscape (moose; Yankee Cosmopolitan) and 94.48% of the landscape (white-tailed deer; Recent Trends; Table 2). That is, for moose < 1% of map cells were persistent under the Yankee Cosmopolitan scenario, whereas for the ubiquitous white-tailed deer almost 95% of map cells were persistent under the Recent Trends scenario. Across scenarios, species with the highest average regional persistence were white-tailed deer (93.25%) and coyote (93.04%), followed by raccoon, striped skunk, and black bear. Wild turkey had the lowest average persistence across the region (7.33%), followed by moose (16.73%), red fox (21.96%), and bobcat (29.22%; Table 2). In terms of the individual scenarios, the percentage of persistent cells across species averaged between 49.61% (Yankee Cosmopolitan) and 56.02% (Growing Global), although the variance in persistence among the species was quite large for each scenario (Table 2).

**Table 2:** Persistence statistics for nine wildlife species in New England, USA.

Species	NELFP scenario simulated persistence (% of region) by year 2060					Average
	Recent Trends	Growing Global	Go It Alone	Yankee Cosmopolitan	Connected Communities	
American black bear	61.64	60.92	66.23	63.20	65.46	63.49
Bobcat	24.30	45.01	23.44	23.97	29.35	29.22
Coyote	93.33	92.03	93.45	92.78	93.63	93.04
Moose	12.07	24.59	28.12	0.99	17.87	16.73
Raccoon	87.03	89.47	87.33	86.41	87.56	87.56
Red fox	21.96	21.91	21.97	21.96	21.99	21.96
Striped skunk	61.49	66.62	61.03	61.22	62.16	62.50
White-tailed deer	94.48	89.89	94.35	93.77	93.75	93.25
Wild turkey	2.67	13.76	14.82	2.17	3.22	7.33
Average	51.00	56.02	54.53	49.61	52.78	

Statistics were derived from scenario simulated distribution change maps and indicate the percent of the New England region where species occurrence is likely to “persist” at an occurrence probability > 0.7 between 2010 and 2060. Persistence statistics were based on species occurrence probabilities under the individual NELFP scenarios.

#### Resistance

**Fig 3:**
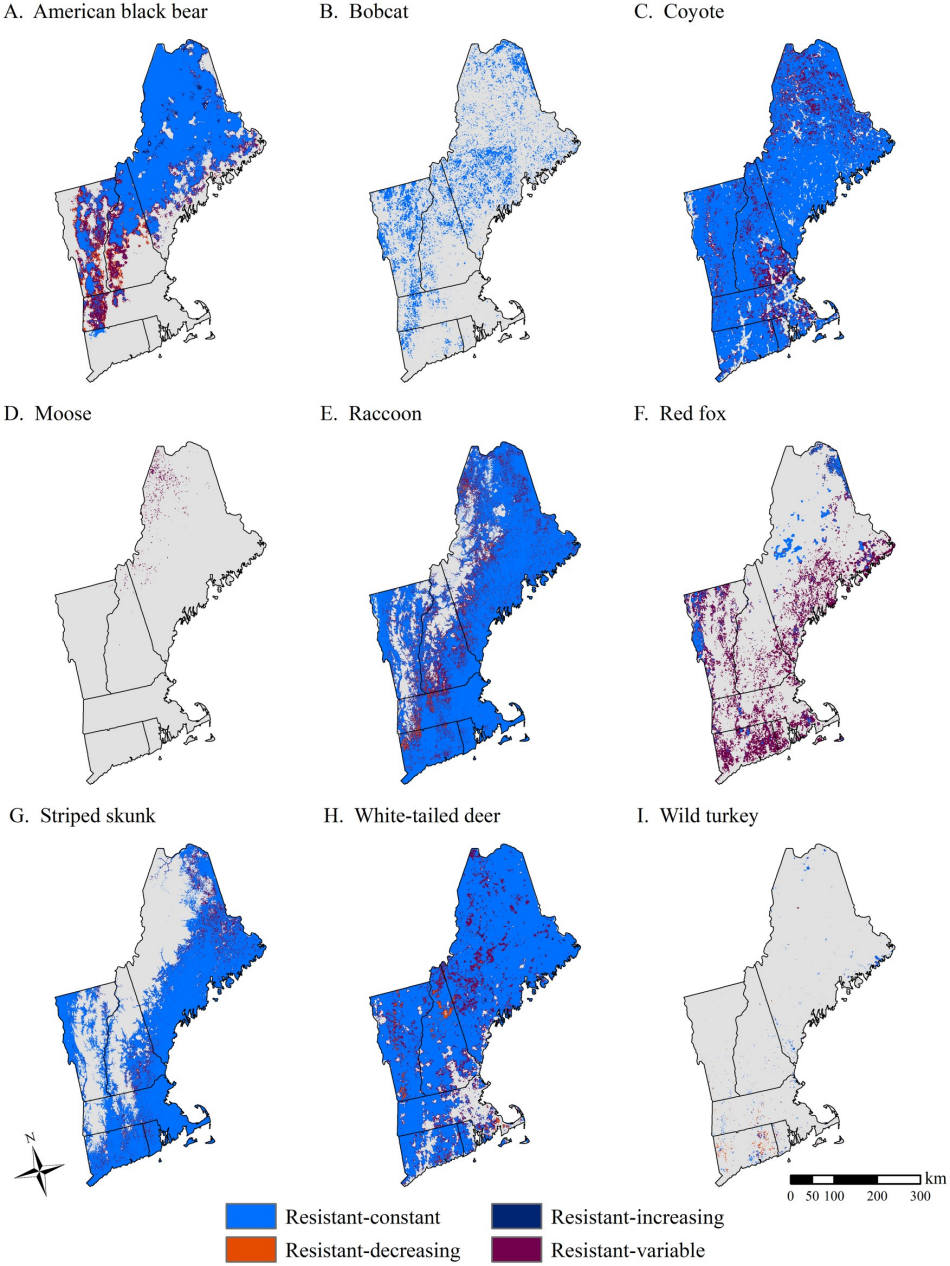
Estimated resistance for nine wildlife species in New England, USA. Resistance was based on scenario projected distribution change between 2010 and 2060. Resistant cells represent areas with high occurrence probabilities (> 0.7) under current conditions *and* across all NELFP scenarios. Resistance is displayed by resistance class: 1) Resistant-constant: occurrence probability remained high (> 0.7) and relatively constant across all scenarios, 2) Resistant-increasing: occurrence probabilities remained high (> 0.7) and increased under all scenarios, 3) Resistant-variable: occurrence probability remained high (> 0.7) but fluctuated among the scenarios, and 4) Resistant-decreasing: occurrence probability remained high (> 0.7) but decreased under all scenarios. Maps correspond with the following species: A) American black bear, B) Bobcat, C) Coyote, D) Moose, E) Raccoon, F) Red fox, G) Striped skunk, H) White-tailed deer, and I) Wild turkey. Map boundary data source: U.S. Census Bureau [29].

Regional resistance—defined as the percentage of cells in the study region that were projected to remain persistent across all 5 future scenarios—was greatest for coyote (91.81%), white-tailed deer (89.72%), raccoon (84.43%), striped skunk (60.81%), and black bear (56.86%; Table 3). Regional resistance was lowest for moose (0.76%), followed by wild turkey (1.30%), bobcat (16.73%), and red fox (21.90%; Table 3; Fig 3).

**Table 3:** Regional resistance statistics for nine wildlife species in New England, USA.

Species	Resistant (%)	Resistant-constant (%)	Resistant-increasing (%)	Resistant-decreasing (%)	Resistant-variable (%)
American black bear	56.86	44.16	0.94	2.06	9.70
Bobcat	16.73	16.73	0.00	0.00	0.00
Coyote	91.81	79.78	0.01	0.01	12.00
Moose	0.76	0.01	0.00	0.01	0.74
Raccoon	84.43	70.62	0.10	2.02	11.70
Red fox	21.90	3.91	0.37	0.02	17.59
Striped skunk	60.81	55.58	0.14	0.14	4.95
White-tailed deer	89.72	77.52	0.36	0.88	10.95
Wild turkey	1.30	0.75	0.01	0.29	0.25

Statistics were derived from scenario simulated distribution change maps and indicate the percent of the New England region where species occurrence is likely to remain “resistant” to change between 2010 and 2060 across *all* NELFP scenarios. Overall resistance was based on species simulated persistence for each NELFP scenario, where persistent pixels had > 0.7 occurrence probability in *both* 2010 and 2060. Resistant pixels were further categorized into resistance classes (constant, increasing, decreasing, and variable) based on change in occurrence probability across the 5 NELFP scenarios.

Within resistant areas, change in occurrence probabilities were generally minimal (< ±0.1). That is, for most species (n = 7) the majority of resistant cells (i.e., > 50%) were classified as resistant-constant (Table 3; Fig 3). Variation in species occurrence probabilities among scenarios was also common; with a moderate level of resistant cells (between 4% and 20%) classified as resistant-variable for all but two species (Table 3; Fig 3). Several species with the low levels of regional resistance—including moose and red fox—experienced high levels of variability in occurrence probability among scenarios (i.e., 97.3% and 80.3% of resistant cells were classified as resistant-variable, respectively; Table 3). For all species, only small portions of resistant cells were consistently increasing (i.e., resistant-increasing) or consistently decreasing (i.e., resistant-decreasing) in occurrence probability across all scenarios (Table 3; Fig 3).

The distribution of resistant areas varied among states, which varied in geographic area (Table 4). For example, coyote was resistant throughout the majority of the region, with 50.84% of the resistant cells occurring in Maine, followed by 15.03% in Vermont and 14.72% in New Hampshire, both of which are geographically smaller (Table 4, Fig 3). However, average species resistance *within* a given state was equally high for Vermont (0.95) and Maine (0.95; Table 4), meaning that 95% of cells in Vermont and 95% of cells in Maine were characterized as resistant for this species. White-tailed deer was resistant throughout large portions of New England, with 54.13% of regional resistance occurring in Maine, and average within-state resistance ranging from 0.60 in Massachusetts to 0.98 in Maine. Raccoon was resistant throughout most of the lower elevation areas in the region (Fig 3). Within states, average resistance ranged from 0.70 in Vermont to 1.00 in Rhode Island, with the relative majority (50.53%) of regional raccoon resistance occurring in Maine. Striped skunk was resistant in low elevation areas throughout much of the region (Fig 3), with highest average resistance in Rhode Island (1.00) and Connecticut (0.89), and the relative majority (48.74%) of regional resistance occurring in Maine. American black bear was predominantly resistant in northern New England, with 68.63% of regional resistance occurring in Maine, and within-state average resistance ranging from 0.00 in Rhode Island to 0.79 in Maine.

**Table 4:** Percentage of regional resistance by state and average resistance within each state for nine wildlife species in New England, USA.

Species	Connecticut		Maine		Massachusetts		New Hampshire		Rhode Island		Vermont	
	Mean	%	Mean	%	Mean	%	Mean	%	Mean	%	Mean	%
American black bear	0.03	0.39	0.79	68.63	0.18	4.07	0.52	13.14	0.00	0.00	0.54	13.77
Bobcat	0.07	3.26	0.18	52.85	0.08	5.92	0.13	11.14	0.01	0.08	0.31	26.75
Coyote	0.85	6.95	0.95	50.84	0.81	11.15	0.93	14.72	0.72	1.30	0.95	15.03
Moose	0.00	0.00	0.01	95.08	0.00	0.00	0.00	4.52	0.00	0.00	0.00	0.40
Raccoon	0.99	8.83	0.86	50.53	0.94	13.96	0.74	12.73	1.00	1.96	0.70	11.98
Red fox	0.45	15.36	0.17	38.32	0.24	13.49	0.13	8.64	0.35	2.62	0.32	21.58
Striped skunk	0.89	11.18	0.60	48.74	0.77	15.96	0.46	11.12	1.00	2.75	0.43	10.25
White-tailed deer	0.78	6.55	0.98	54.13	0.60	8.44	0.88	14.20	0.66	1.22	0.95	15.46
Wild turkey	0.07	37.79	0.01	33.46	0.02	15.79	0.00	4.87	0.04	5.22	0.00	2.87
Average	0.46	10.03	0.51	54.73	0.40	9.86	0.42	10.56	0.42	1.68	0.47	13.12

Statistics were calculated from species binary resistance maps developed for the region and provide measures for 1) Mean resistance: the proportion of the state where species occurrence is resistant to change between 2010 and 2060, and 2) Percent of regional resistance: the percentage of a species regional resistance that occurs within each state.

For the species with lower average regional resistance—including red fox, bobcat, wild turkey, and moose (Table 2)–resistant areas were generally smaller and less connected. The resistant red fox cells occurred in moderately sized patches throughout New England (Fig 3). Average within-state resistance was highest in Connecticut (0.45) and Rhode Island (0.35) while average regional resistance was highest in Maine (38.32%) and Vermont (21.58%). The resistant cells for bobcat were dispersed in moderate-small patches throughout the region, with the majority (52.85%) of regional resistance occurring in Maine and the highest within-state average resistance (0.31) occurring in Vermont (Table 4, Fig 3). Wild turkey was resistant in small patches throughout New England, with both the relative majority (37.79%) of regional resistance and the highest within-state average resistance (0.07) occurring in Connecticut. Moose resistance was extremely low throughout New England, with the vast majority of resistant cells occurring in Maine and New Hampshire. Within states, resistant cells occurred in 1.50% of Maine, 0.24% of New Hampshire and 0.02% of Vermont (average within-state resistance of 0.0150, 0.0024, and 0.0002, respectively; Table 4, Fig 3).

### Objective 3—Protected areas

New England’s protected area network currently contains 57,449 protected parcels—including federal, state, and municipal parcels and others managed by non-profits (e.g., The Nature Conservancy) [28]. In 2010, most of the region’s protected areas (54.12%) were under public ownership (e.g., White Mountain National Forest), held as private lands under protective easements (32.45%), or were protected under non-profit ownership (11.70%) [38]. The size of individual PAs varied significantly, with parcels sizes ranging from < 1 km^2^ to 3047.65 km^2^. Protected parcels in the more rural northern portion of New England were generally larger than parcels in the southern states; with land conservation in Connecticut, Rhode Island, and Massachusetts characterized by numerous small parcels no larger than 40.47 km^2^ (10,000 acres) [38]. Parcel protection also varied in land-use restrictions. For example, many PAs allowed timber harvesting but did not allow land conversion (e.g., forest to development) [38]. Overall, approximately 22% of the New England region was under some form of land protection.

Given the size of the region and the existing protected network, only small portions of the region were both protected and resistant for individual species (Table 5; column 3). For example, 91.81% of the map cells in New England were classified as resistant for coyote (i.e., marginal probability of resistance = 0. 9181), but only 21.12% of the resistant cells were also protected (i.e., joint probability of resistance & protection = 0.2112). Resistance of other species is even less protected under the current protected network. For example, of the 16.73% of the region that was classified as resistant for bobcat, only 3.26% is currently protected (Table 2; Table 5, columns 2 & 3). Large portions of the region were both unprotected and not resistant for many species. For example, nearly all of New England’s unprotected land (approximately 78% of the region) was also not resistant for moose (0.7763) and wild turkey (0.7724; Table 5, column 4). Bobcat and red fox also simulated large portions of the region that were neither protected nor resistant (i.e., joint probability—unprotected & not resistant = 0.6478 and 0.5916, respectively; Table 5, column 4).

**Table 5:** Protected area resistance statistics for nine wildlife species in New England, USA.

Species	Marginal probability of resistance	Joint probability: resistant & protected	Joint probability: not resistant & unprotected	Conditional probability of protection given resistance	Conditional probability of resistance given protection
American black bear	0.5686	0.1554	0.3686	0.2734	0.7123
Bobcat	0.1673	0.0326	0.6478	0.1945	0.1497
Coyote	0.9181	0.2112	0.0753	0.2300	0.9694
Moose	0.0076	0.0025	0.7763	0.3328	0.0115
Raccoon	0.8443	0.1454	0.0835	0.1723	0.6683
Red fox	0.2190	0.0274	0.5916	0.1252	0.1265
Striped skunk	0.6081	0.1415	0.3131	0.2328	0.6422
White-tailed deer	0.8972	0.2087	0.0945	0.2326	0.9618
Wild turkey	0.0130	0.0038	0.7724	0.2890	0.0173

All statistics were calculated using species binary resistance maps developed for the region and polygons from the Protected Areas Database of the U.S. (PAD-US version 2.0) [28]. Statistics include 1) Marginal probability of resistance: the proportion of the region that is resistant for each species, 2) Joint probability—resistant and protected: the proportion of the region that is both protected and resistant, 3) Joint probability—not resistant and unprotected: the proportion of the region that is unprotected and does not meet the resistance criteria, 4) Conditional probability of protection given resistance: the proportion of each species regional resistance that is protected, and 5) Conditional probability of resistance given protection: the proportion of the protected network that is resistant for each species. Note that the protected network covers 21.63% of the New England region (i.e., marginal probability of protection = 0.2163).

The relationship between resistance and protection can be expressed from different points of view. The conditional probability of protection, given a species resistance, is the proportion of a species resistant cells that are also protected (Table 5, column 5). For most species the protected network encompassed moderate levels of the species regional resistance. That is, for all but three species (red fox, raccoon, and bobcat), between 20% and 35% of the resistant cells were also protected (Table 5; column 5). Conditional probability of protection (given the species resistance), was highest for moose (0.3328), followed by wild turkey (0.2890), and black bear (0.2734; Table 5, column 5). White-tailed deer, coyote, and striped skunk experienced moderate-to-low levels of regional resistance protection with conditional probabilities ranging between 0.2326 and 0.2300 (Table 5).

The relationship between resistance and protection can also be viewed from the perspective of the protected network. Given the region’s protected cells, we can determine the proportion of the protected network that is also resistant for each species (Table 5, column 6). Resistance was well represented within the current protected network for some focal species and poorly represented for others. Coyote, white-tailed deer, black bear, raccoon, and striped skunk had the highest representation of resistance in protected areas (Table 5, column 6). For coyote, the conditional probability of resistance occurring within protection was 0.9694 –indicating that 96.94% of the protected maps cells in New England were designated as resistant. White-tailed deer (0.9618) and black bear (0.7123) also had relatively high conditional probability of resistance (given protection), while moose (0.0115) and wild turkey (0.0173) had very low representation of resistant cells within the protected network, thus low conditional probabilities (Table 5).

**Fig 4:**
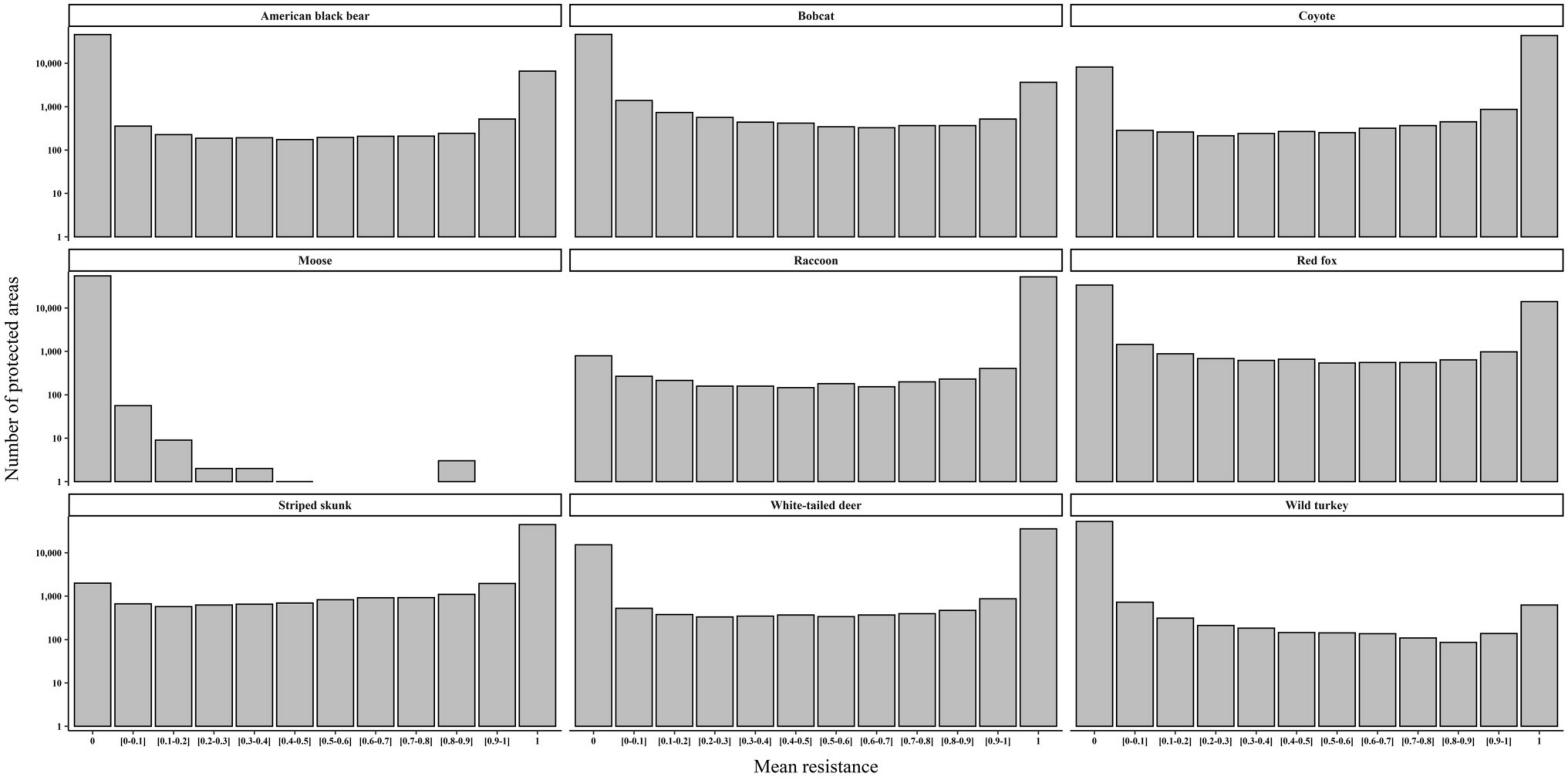
Focal species resistance within New England’s protected areas. Mean resistance indicates the proportion of cells in a protected parcel that are resistant for a given species. Graphs display trends in species mean resistance within individual parcels. Note the logarithmic scale of the y-axes.

The relationship between species resistance and protection was also evaluated for individual PAs within the protected network. Average species resistance *within* individual PAs ranged between 0 and 1 (Fig 4). However, for most PAs average species resistance was either 0 or 1 –meaning that most PAs were either fully resistant (i.e., all cells in the PA were resistant for the target species) or contained zero resistant cells for a given species (Fig 4; note the log scale). For example, the majority (~80%) of the region’s PAs (i.e., 45968 PAs) had an average resistance of 0 for black bear. However, the PAs that did contain > 0 average resistance for black bear were often fully resistant (i.e., average resistance was 1 for 6596 PAs; Fig 4). For some of the species—including red fox, moose, wild turkey, and bobcat—the majority of PAs had an average resistance of 0. However, for other species—including raccoon, striped skunk, coyote, and white-tailed deer—the majority of the region’s PAs were fully resistant.

**Fig 5:**
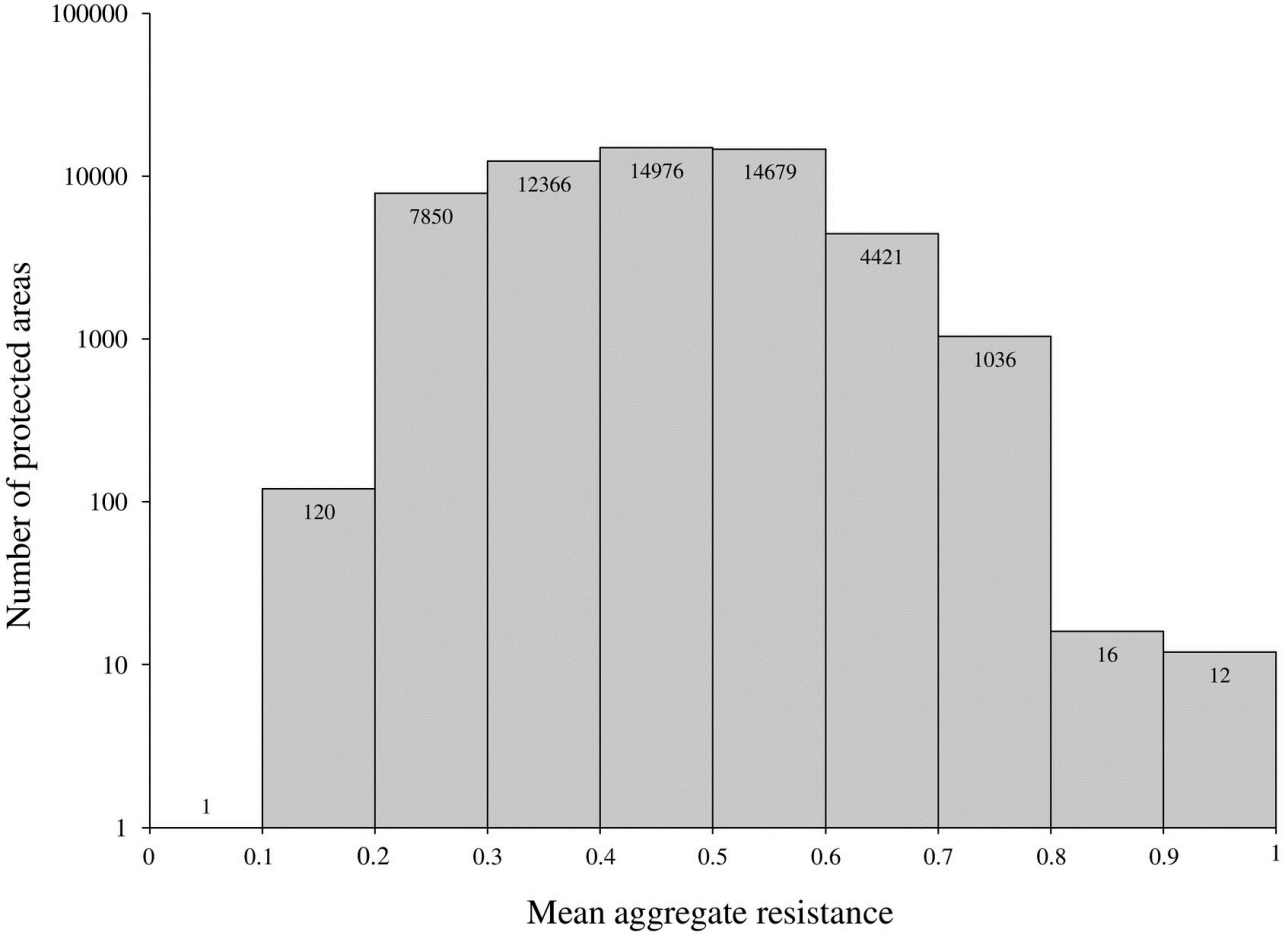
Aggregate focal species resistance within New England’s protected areas. Mean aggregate resistance provides a standardized indicator of resistance for each protected parcel based on the average focal species resistance within the parcel. Graph displays trends in mean aggregate resistance within individual parcels.

Aggregate focal species resistance was evaluated across the region and within individual PAs. Aggregate focal species resistance was calculated for each cell in the region as the average binary resistance across the 9 species. Thus, an aggregate resistance of 0 indicated that none of the 9 focal species were resistant, while an aggregate resistance of 1 indicated all 9 species were resistant. Mean aggregate resistance was computed for each PA by averaging the aggregate resistance values. This generated a comparable resistance score for all PAs in the protected network. Spatial variability in species resistance lead to generally moderate mean aggregate resistance throughout the region (regional mean aggregate resistance = 0.4716; S2 Fig) and the protected network (Fig 5). The majority of the region’s PAs (~64%) had mean aggregate resistance scores below 0.5 (Fig 5; note the log scale). For example, 120 PA’s had a mean aggregate resistance between 0.1 and 0.2. In contrast, 28 PAs had a mean aggregate resistance > 0.8; indicating that these PAs provided high levels of resistance protection for the majority of the focal group (Fig 5).

## Discussion

Identifying areas of resistance for wildlife represents a conservation priority, especially in the New England region, which is experiencing rapid climate and land-use changes [17,21,23]. We evaluated how the distributions of nine focal species are expected to change in response to 50-years of climate change and alternative land-use trajectories. We assessed cross-scenario trends in species resistance to identify areas where species exhibited the greatest resistance to future disturbances and analyzed how species spatial resistance aligned with the current protected area network. Our analyses provide a new approach for evaluating species spatial resistance, generate questions about the long-term success of harvested species in the New England region, and highlight the value and utility of scenario-based species resistance assessments for conservation planning.

### Scenario-based resistance

Our scenario results reinforce the belief that future changes in climate and land use will likely have variable and often negative consequences for wildlife species in the New England region. Spatial patterns in species occurrence and regional resistance varied considerably among the focal group, which reflects the diverse habitat requirements of the species selected [57]. Overall, species with more general habitat requirements and lower sensitivity to climate or development—including coyote, white-tailed deer, raccoon, and striped skunk—exhibited the highest levels of occurrence stability and regional resistance. Alternatively, species with narrower habitat requirements and higher sensitivity to landscape change, such as moose and wild turkey, exhibited low regional resistance, meaning that few cells that were high-quality under the baseline projection remained high-quality under all 5 scenarios considered. For these low resistance species, the small number of cells that are resistant may be of high conservation value—providing high-quality habitat that is robust to future change.

Two species projections merit special discussion. First, for species such as red fox, low regional resistance does not necessarily mean this species is at risk. For example, red fox will likely occupy considerable portions of a future New England landscape. However, due to generally moderate occurrence probability under current conditions and climate-related increases in occurrence probability under future conditions, only small portions of the region were considered resistant to change. In the context of this study, resistant map cells designate locations where species have high occurrence probability under current conditions that remain high despite uncertain future conditions. It is important to recognize that we expect wildlife species to occur outside of these resistant areas in the future; however, species are not necessarily resistant to changes that may occur in these areas. Second, moose exhibited extremely low cross-scenario resistance, and significant variation in scenario-specific persistence. For example, under the Go It Alone scenario, 28.12% of the region represented persistent areas for moose. However, under the Yankee Cosmopolitan scenario only 0.99% of the region was persistent for moose. This suggests that moose will experience considerably higher levels of resistance if New England undergoes changes similar to that of the Go It Alone scenario (in which heavily managed forests will benefit moose), rather than the Yankee Cosmopolitan scenario. For species like moose, land use planning is particularly important because different futures could result in very different distribution and resistance patterns.

### Implications for conservation

With spatial heterogeneity in environmental change and species responses to change, spatially explicit approaches to management and conservation are increasingly necessary [2–4]. Our approach provides spatially explicit quantitative information about species occurrence that can help guide management and land use decisions at multiple spatial and temporal scales. Because state governments typically regulate wildlife management and harvest decisions in the US, state-level resistance statistics can help guide species management and the allocation of limited funds. Understanding which species are most resistant to change or vulnerable to decline within a given state can also inform state-based planning and help ensure that both state and regional conservation objectives are being met. Species-specific resistance maps can help decision-makers identify locations for conservation activities as well as sites potentially suited for non-wildlife related resource management, or development. Obtaining this information for multiple species and at a regional scale can provide a basis for directing limited resources to areas where they are most beneficial to broad-scale conservation [3,4,66].

Understanding which species are likely to remain well represented in the protected network and which species may become more reliant on PAs may be particularly useful information for evaluating representation and persistence targets within existing PAs, and for identifying gaps in the current network [67]. We found that species with higher levels of regional resistance—including coyote, white-tailed deer, raccoon, striped skunk, and black bear—were generally well represented in the protected network. This means that the current protected network is likely to conserve the focal species that have the highest resistance overall. However, for species with low levels of regional resistance—including moose and wild turkey—the conditional probability of resistance within protected areas was not negligible (e.g., 33% for moose). That is, of the few resistant pixels for moose, 33% are under some form of protection. This indicates that protected areas may be particularly important to the future success of these species. For these low resistance species, the few areas that are resistant-constant or resistant-increasing may be particularly valuable sites for conservation. By adding a species’ resistant sites to the protected network, these areas may be able to host source populations that can sustain less productive areas within the region and contribute to species persistence [68].

Conservation strategies for large, fragmented, and rapidly changing regions need to prioritize areas where conservation targets are most likely to persist long-term [67,69]. Spatial prioritization tools, such as Marxan [70] and Zonation [71], have been developed to help identify potential reserve sites that satisfy regional conservation goals. These computational decision-support tools can guide the design of protected areas and reserve systems when complex trade-offs exist [72,73]. However, the successful application of these tools requires reliable information about species distributions and long-term persistence [69]. Our results satisfy these requirements by providing fine-scale species occurrence and resistance information in a regional context. Our models estimate occurrence on a cell-by-cell basis by evaluating covariates at spatial scales relevant to the focal species. This process generates fine-resolution tools that account for broader species-scale influences and are compatible with existing spatial prioritization methods. While individual (30 x 30 m) map cells are often not sufficient targets for conservation, fine-resolution maps provide detailed metrics that can guide the selection of larger parcels and help prioritize species or locations that may require conservation attention.

With increasing environmental change, maintaining or improving connectivity within regional landscapes is often a conservation priority to allow for gene flow and support population growth [9,74,75]. Spatial resistance maps can help identify potential pathways for connectivity among resistant areas and throughout landscapes [9]. In human-dominated landscapes, habitat connectivity can facilitate movement of individuals (and their genes), which supports larger population sizes and reduces potential isolation and related demographic and genetic consequences [76–78]. Through the combined utility of SDMs and alternative scenarios, our maps provide a means of identifying optimal ways to connect critical natural areas and protect species despite an uncertain future.

We suggest that spatially explicit species resistance tools facilitate planning by providing the ability to locate areas where conservation actions are likely to have the most significant long-term benefits for wildlife species. This study provides insight into the spatial consequences of future change for wildlife species, advances our understanding of resistance at multiple spatial and ecological scales, and can help guide reserve design and conservation actions that ensure the longevity of natural systems.

### Caveats to interpretation

Although our study provides novel information about species resistance in an uncertain future, there are limitations to the results that should be considered. First, resistance is a complex concept often focused on numerous ecological functions (e.g., [2,4,10,79,80]). Many studies evaluate resistance through broader conceptual methods, but here we aimed to quantify the spatial resistance of individual wildlife species. Because this approach only targets resistance at the species level, we do not directly address the complexities of ecological resistance, nor do we focus on ecosystem or species interactions. We also acknowledge that there is uncertainty in the models and parameters that simulate species occurrence, and that this approach assumes that relationships between landscape factors and occurrence will remain constant (i.e., species distributions will be driven by the same effects over time).

Second, because our focus is on maintenance of high quality pixels for individual species, cells were only designated as resistant if species occurrence was high in the baseline projection at year 2010 and remained high in the scenario projections at year 2060. In this approach, only map cells that started and remained above the high occurrence threshold (0.7) across all NELFP scenarios were considered resistant; which in some cases excluded maps cells that had high occurrence probabilities at year 2060 but missed the threshold under current conditions. For alternative assessments, it may be important to acknowledge these high occurrence areas as they provide additional information about species local and regional representation. However, for this assessment we targeted areas of persistence to identify the locations where species occurrence is most resistant despite future change. Our persistence (and subsequently resistance) calculations and conclusions are dependent upon the mathematically assumptions used. Given our raster layers for each species and NELFP scenario, it would be straightforward for future research to apply different persistence or constancy criteria to evaluate resistance for alternate objectives.

Third, we acknowledge that there is uncertainty in the models and parameters that simulated species occurrence, land-use change, and forest growth for each scenario. We used species distribution models that performed well when tested against empirical data [62]; however, there is inherent uncertainty in all probability estimates and future simulations. We also acknowledge that New England may change in ways outside the scope of the NELFP scenarios. While we are unable to consider all possible futures, the NELFP scenarios capture relevant uncertainties about the region’s future landscape conditions. The central idea of scenario-planning is to consider a variety of possible futures that include many important elements of uncertainty rather than focusing on the accurate prediction of a single outcome [32]. Our approach builds from this concept and aims to overcome uncertainty about wildlife futures by identifying areas of greatest resistance across multiple scenarios. This approach is not intended as an alternative to other studies. Rather, our results are meant to complement the work of others by providing new scenario-based perspectives and spatially explicit information for individual species. Despite their limitations, these tools have considerable value and can be used alongside other tools and reserve design methods to evaluate the ecological impacts of management decisions and help inform effective long-term conservation.

## Supporting information

S1 Table Distribution change statistics for nine wildlife species in the New England region of the northeastern United States. Statistics were calculated from scenario-simulated distribution change maps (2010–2060) derived from species-specific distribution models developed by [62] and landscape change scenarios developed by the New England Landscape Futures Project [31,58].
(PDF)

S1 Fig Species scenario-specific distribution change throughout New England, USA. Distribution change was projected for nine wildlife species between current (2010) conditions and each of the NELFP scenarios: A) Business-As-Usual, B) Connected Communities, C) Yankee Cosmopolitan, D) Go It Alone, and E) Growing Global. Maps display changes in species probability of occurrence, derived from simulated distribution maps for 2010 and 2060 (see [34,62] for more details).
(PDF)

S2 Fig Aggregate focal species resistance throughout New England, USA. Map displays mean species resistance between 2010 and 2060 based on binary resistance maps for nine focal wildlife: American black bear, bobcat, coyote, moose, raccoon, red fox, striped skunk, white-tailed deer, and wild turkey.
(PDF)
