# Supplementary material for: Wildlife resistance and protection in a changing New England landscape
Source: PLoS One. 2020 Sep 24;15(9):e0239525. doi: 10.1371/journal.pone.0239525 (PMC7515594; doi:10.1371/journal.pone.0239525)
Supplement: S1 Table — Statistics were calculated from scenario-simulated distribution change maps (2010–2060) derived from species-specific distribution models developed by [62] and landscape change scenarios developed by the New England Landscape Futures Project [31,58]. (PDF) [file pone.0239525.s001.pdf]

S1 Table. Distribution change statistics for nine wildlife species in the New England region of the northeastern United States. Statistics were calculated from scenario-simulated distribution change maps (2010 – 2060) derived from species-specific distribution models developed by [62] and landscape change scenarios developed by the New England Landscape Futures Project [31,58].

Species	Scenario	Mean	Standard deviation	Minimum	Quartiles			Maximum
					25%	50%	75%	
American black bear	RT	-0.12	0.22	-0.94	-0.20	-0.05	0.00	0.82
	GG	-0.15	0.25	-0.96	-0.25	-0.05	0.00	0.79
	GA	-0.09	0.19	-0.93	-0.13	-0.03	0.01	0.81
	YC	-0.14	0.23	-0.95	-0.21	-0.05	0.00	0.83
	CC	-0.11	0.20	-0.93	-0.18	-0.04	0.00	0.81
Bobcat	RT	-0.04	0.07	-0.65	-0.08	-0.03	0.01	0.41
	GG	-0.04	0.11	-0.66	-0.06	0.00	0.03	0.39
	GA	-0.04	0.07	-0.65	-0.08	-0.03	0.01	0.41
	YC	-0.05	0.08	-0.53	-0.09	-0.03	0.00	0.38
	CC	-0.02	0.07	-0.59	-0.06	-0.01	0.02	0.44
Coyote	RT	-0.03	0.04	-0.32	-0.04	-0.02	0.00	0.57
	GG	-0.02	0.05	-0.38	-0.03	-0.01	0.00	0.76
	GA	-0.03	0.04	-0.37	-0.04	-0.02	0.00	0.39
	YC	-0.03	0.04	-0.37	-0.05	-0.02	-0.01	0.39
	CC	-0.03	0.04	-0.37	-0.04	-0.01	0.00	0.40
Moose	RT	-0.27	0.19	-0.94	-0.42	-0.27	-0.10	0.87
	GG	-0.15	0.18	-0.94	-0.27	-0.11	-0.01	0.70
	GA	-0.10	0.14	-0.94	-0.19	-0.07	0.00	0.73
	YC	-0.33	0.19	-0.94	-0.47	-0.35	-0.18	0.74
	CC	-0.22	0.18	-0.94	-0.36	-0.21	-0.07	0.76
Raccoon	RT	-0.05	0.05	-0.37	-0.08	-0.04	0.00	0.43
	GG	-0.02	0.06	-0.36	-0.05	-0.01	0.02	0.51
	GA	-0.05	0.05	-0.37	-0.08	-0.04	-0.01	0.44
	YC	-0.05	0.06	-0.37	-0.08	-0.04	0.00	0.43
	CC	-0.05	0.05	-0.35	-0.08	-0.04	-0.01	0.43
Red fox	RT	0.19	0.08	-0.46	0.15	0.19	0.22	0.72
	GG	0.19	0.08	-0.42	0.16	0.20	0.23	0.68
	GA	0.19	0.08	-0.45	0.15	0.19	0.22	0.72
	YC	0.19	0.08	-0.43	0.15	0.19	0.22	0.72
	CC	0.19	0.08	-0.46	0.15	0.20	0.22	0.71
Striped skunk	RT	-0.04	0.06	-0.48	-0.08	-0.04	0.00	0.63
	GG	0.00	0.05	-0.45	-0.03	0.00	0.02	0.67
	GA	-0.05	0.06	-0.54	-0.08	-0.04	0.00	0.64
	YC	-0.05	0.06	-0.54	-0.08	-0.04	0.00	0.64
	CC	-0.04	0.06	-0.46	-0.07	-0.03	0.00	0.64
White-tailed deer	RT	0.00	0.08	-0.85	-0.03	0.00	0.02	0.91
	GG	-0.04	0.10	-0.88	-0.06	-0.02	0.01	0.87
	GA	-0.02	0.09	-0.85	-0.06	-0.03	0.00	0.91
	YC	0.00	0.08	-0.85	-0.03	0.00	0.02	0.91
	CC	-0.01	0.07	-0.85	-0.04	-0.01	0.01	0.92
Wild turkey	RT	-0.16	0.16	-0.94	-0.26	-0.15	-0.06	0.80
	GG	-0.11	0.18	-0.95	-0.21	-0.08	0.01	0.85
	GA	-0.15	0.16	-0.94	-0.25	-0.14	-0.05	0.81
	YC	-0.17	0.16	-0.95	-0.26	-0.16	-0.06	0.81
	CC	-0.16	0.16	-0.94	-0.25	-0.15	-0.05	0.81
