# Supplementary material for: Wildlife resistance and protection in a changing New England landscape
Source: PLoS One. 2020 Sep 24;15(9):e0239525. doi: 10.1371/journal.pone.0239525 (PMC7515594; doi:10.1371/journal.pone.0239525)

S1 Fig. Species scenario-specific distribution change throughout New England, USA. Distribution change was projected for nine wildlife species between current (2010) conditions and each of the NELFP scenarios: A) Business-As-Usual, B) Connected Communities, C) Yankee Cosmopolitan, D) Go It Alone, and E) Growing Global. Maps display changes in species probability of occurrence, derived from simulated distribution maps for 2010 and 2060 (see [34,62] for more details).

A. American black bear

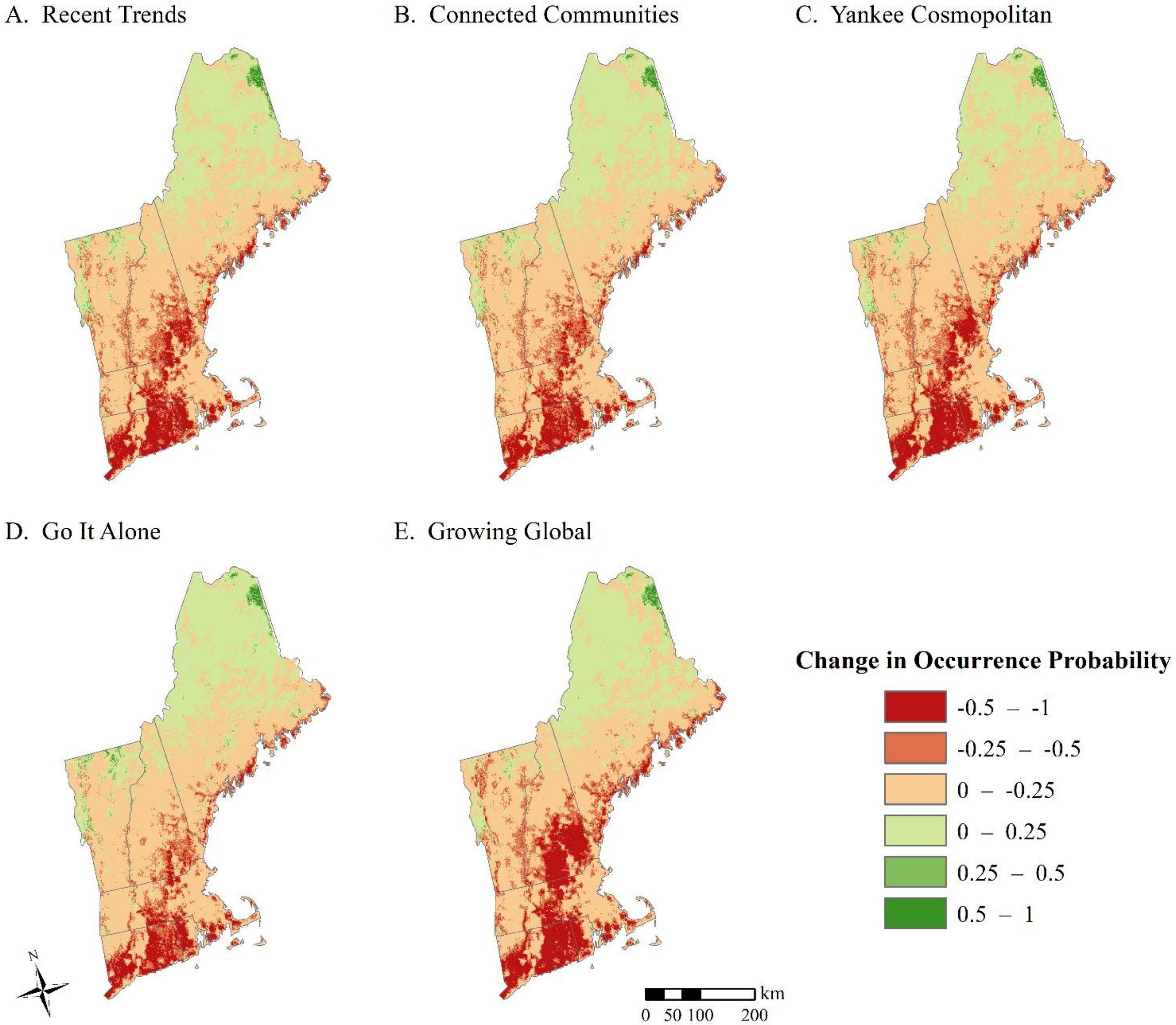

B. Bobcat

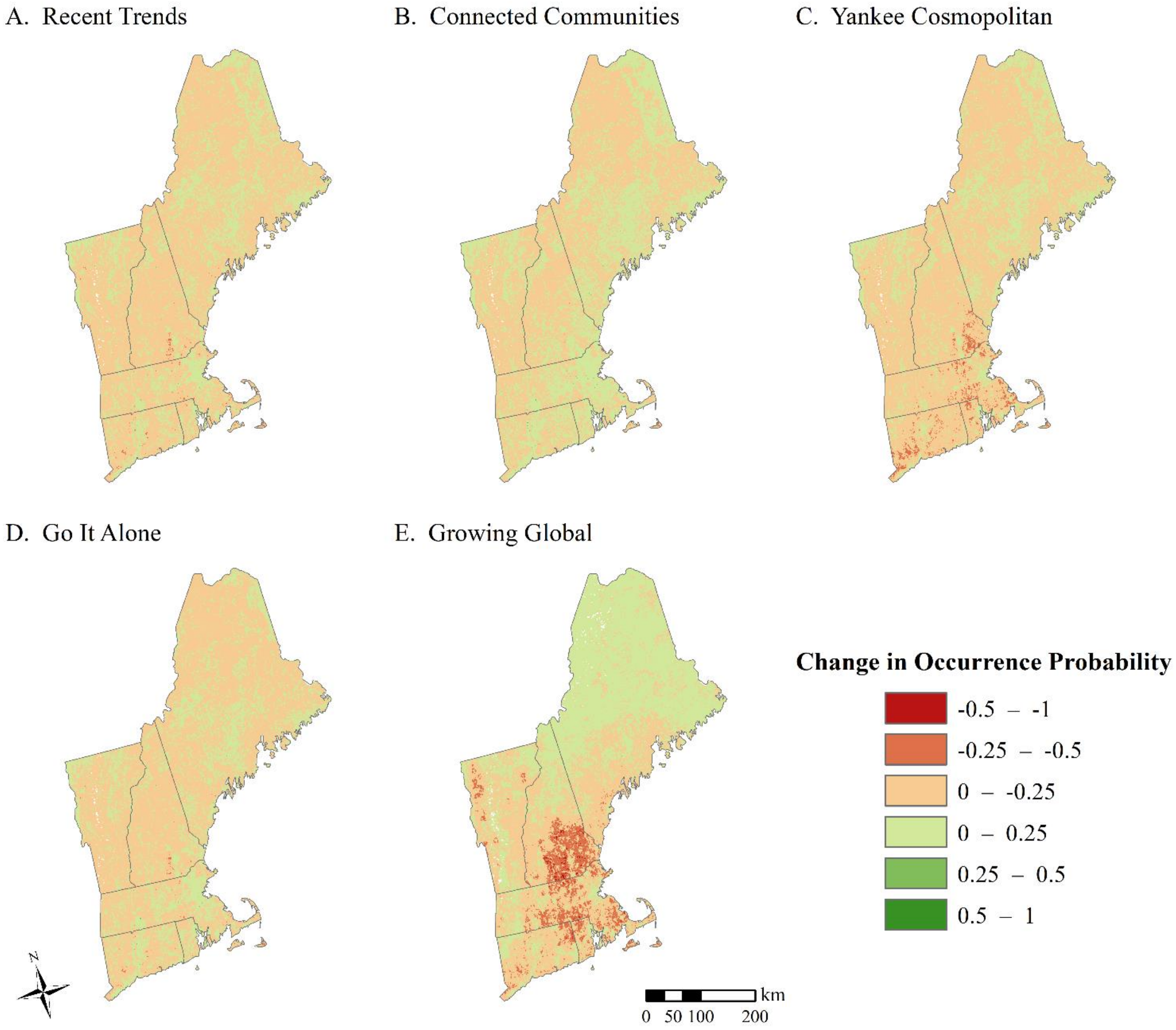
A. Recent Trends
B. Connected Communities
C. Yankee Cosmopolitan
D. Go It Alone
E. Growing Global
Change in Occurrence Probability
-0.5 – -1
-0.25 – -0.5
0 – -0.25
0 – 0.25
0.25 – 0.5
0.5 – 1
N
km
0 50 100 200

C. Coyote

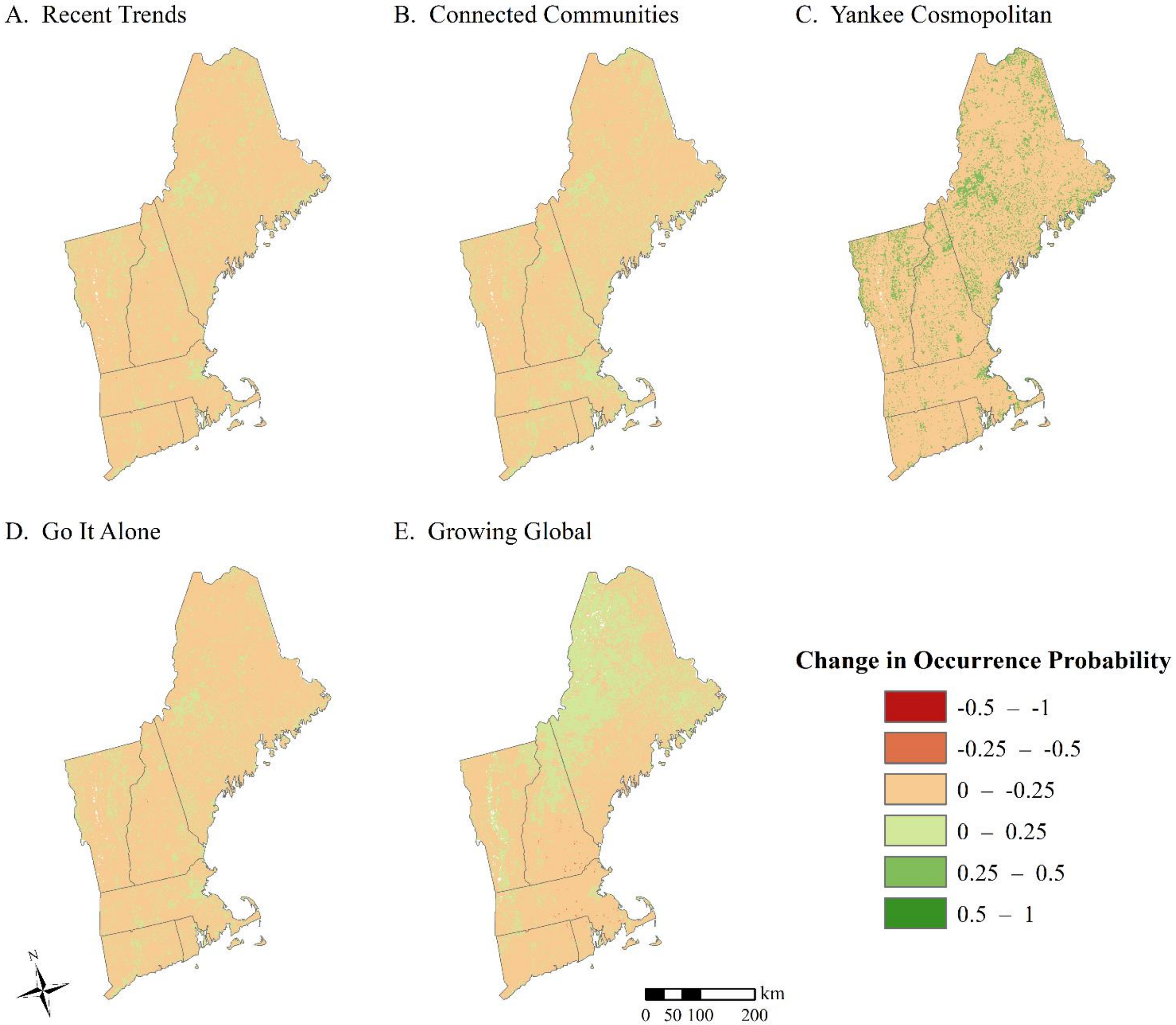
A. Recent Trends
B. Connected Communities
C. Yankee Cosmopolitan
D. Go It Alone
E. Growing Global
Change in Occurrence Probability
-0.5 – -1
-0.25 – -0.5
0 – -0.25
0 – 0.25
0.25 – 0.5
0.5 – 1
N
km
0 50 100 200

D. Moose

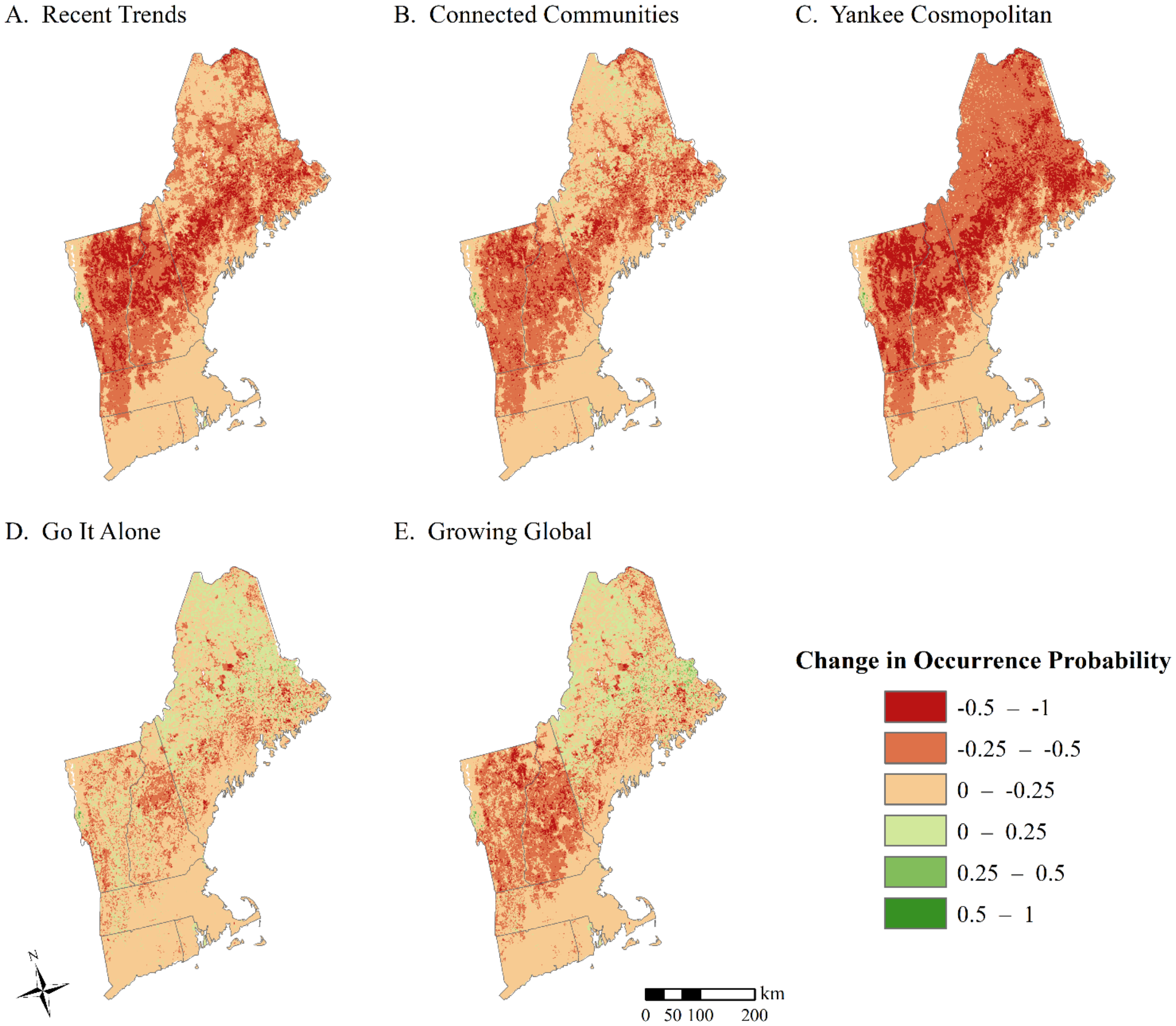
A. Recent Trends
B. Connected Communities
C. Yankee Cosmopolitan
D. Go It Alone
E. Growing Global
Change in Occurrence Probability
-0.5 – -1
-0.25 – -0.5
0 – -0.25
0 – 0.25
0.25 – 0.5
0.5 – 1
N
km
0 50 100 200

E. Raccoon

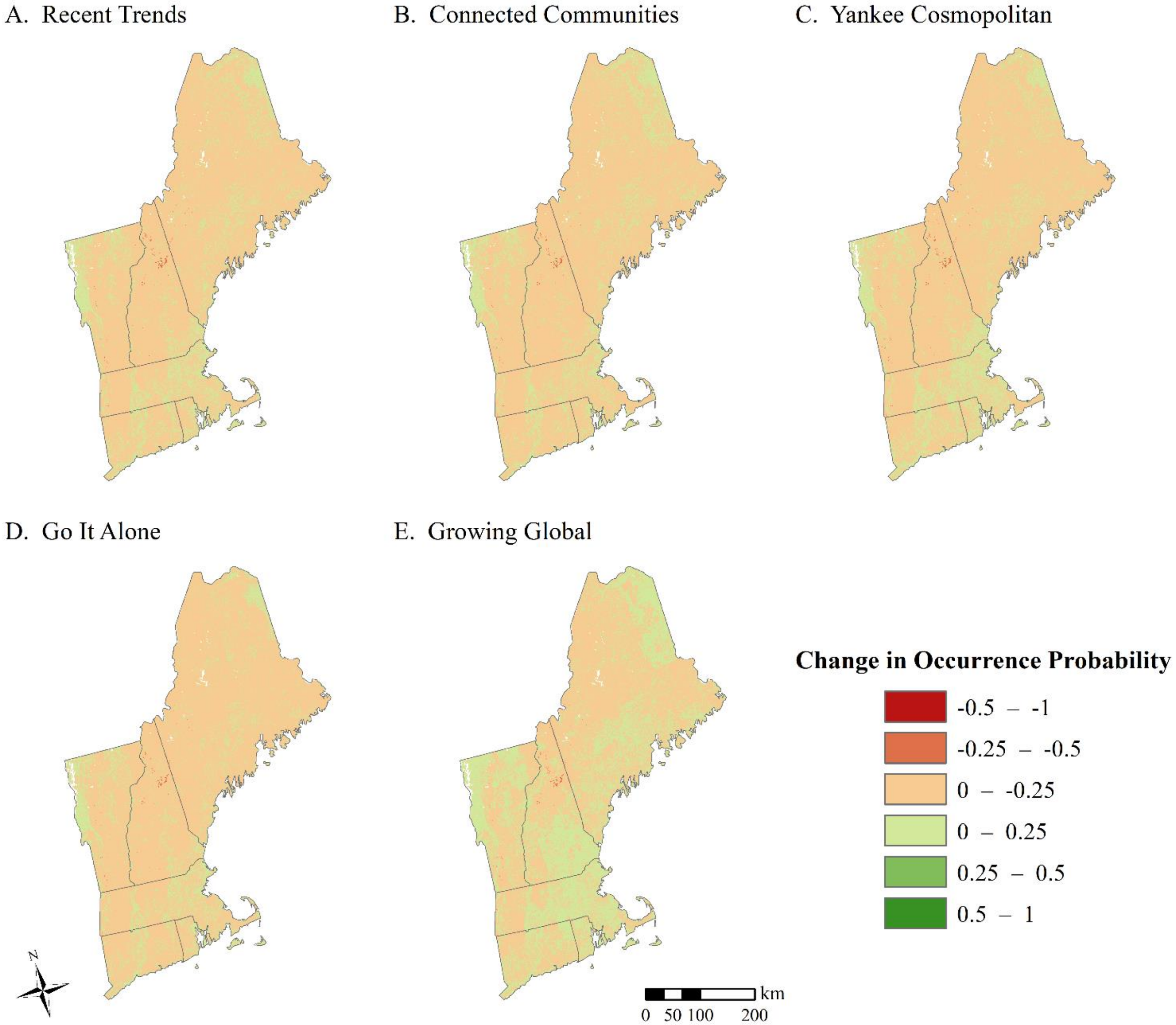

A. Recent Trends
B. Connected Communities
C. Yankee Cosmopolitan
D. Go It Alone
E. Growing Global
Change in Occurrence Probability
-0.5 – -1
-0.25 – -0.5
0 – -0.25
0 – 0.25
0.25 – 0.5
0.5 – 1
N
km
0 50 100 200

F. Red fox

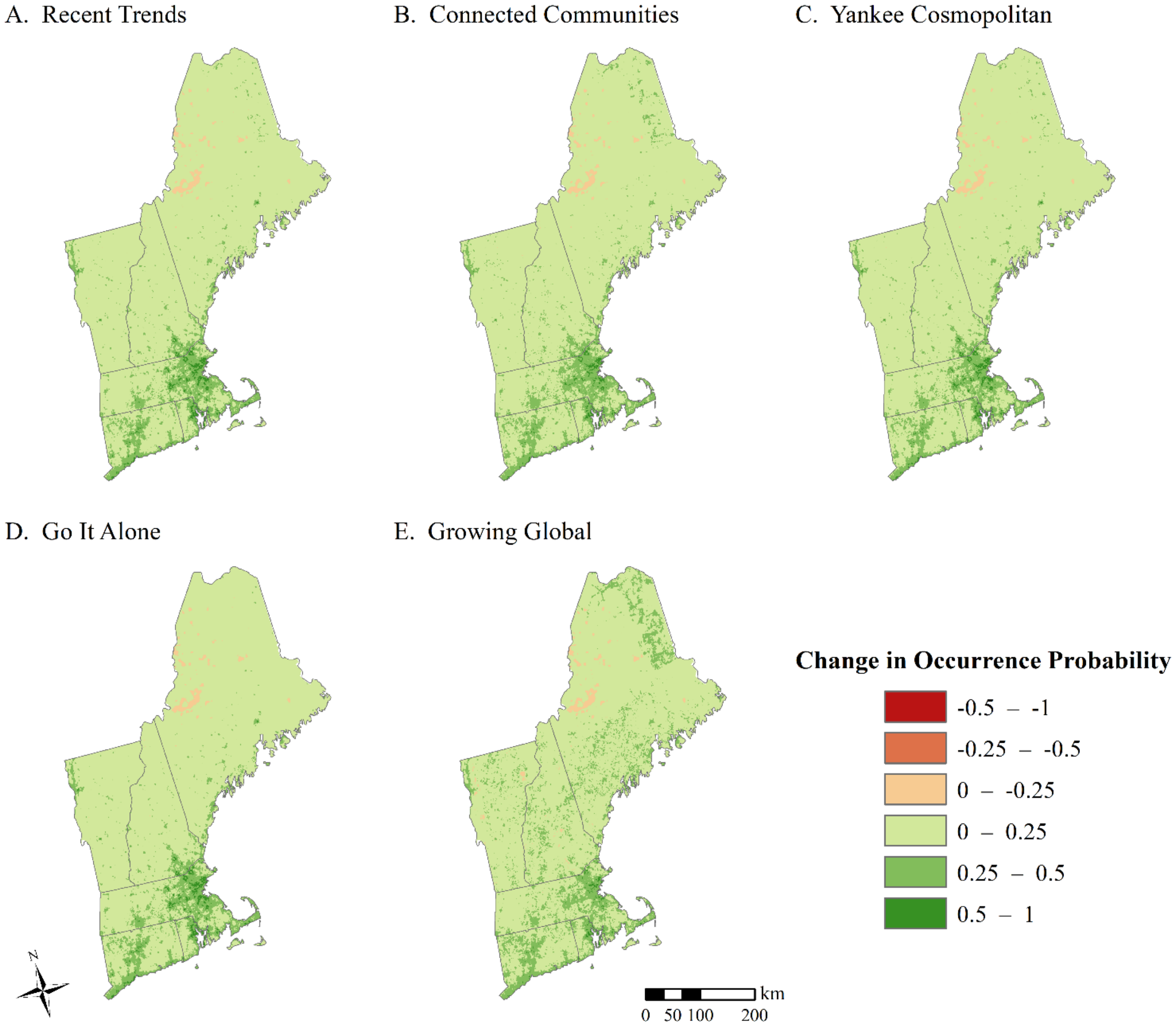
A. Recent Trends
B. Connected Communities
C. Yankee Cosmopolitan
D. Go It Alone
E. Growing Global
Change in Occurrence Probability
-0.5 – -1
-0.25 – -0.5
0 – -0.25
0 – 0.25
0.25 – 0.5
0.5 – 1
N
km
0 50 100 200

G. Striped skunk

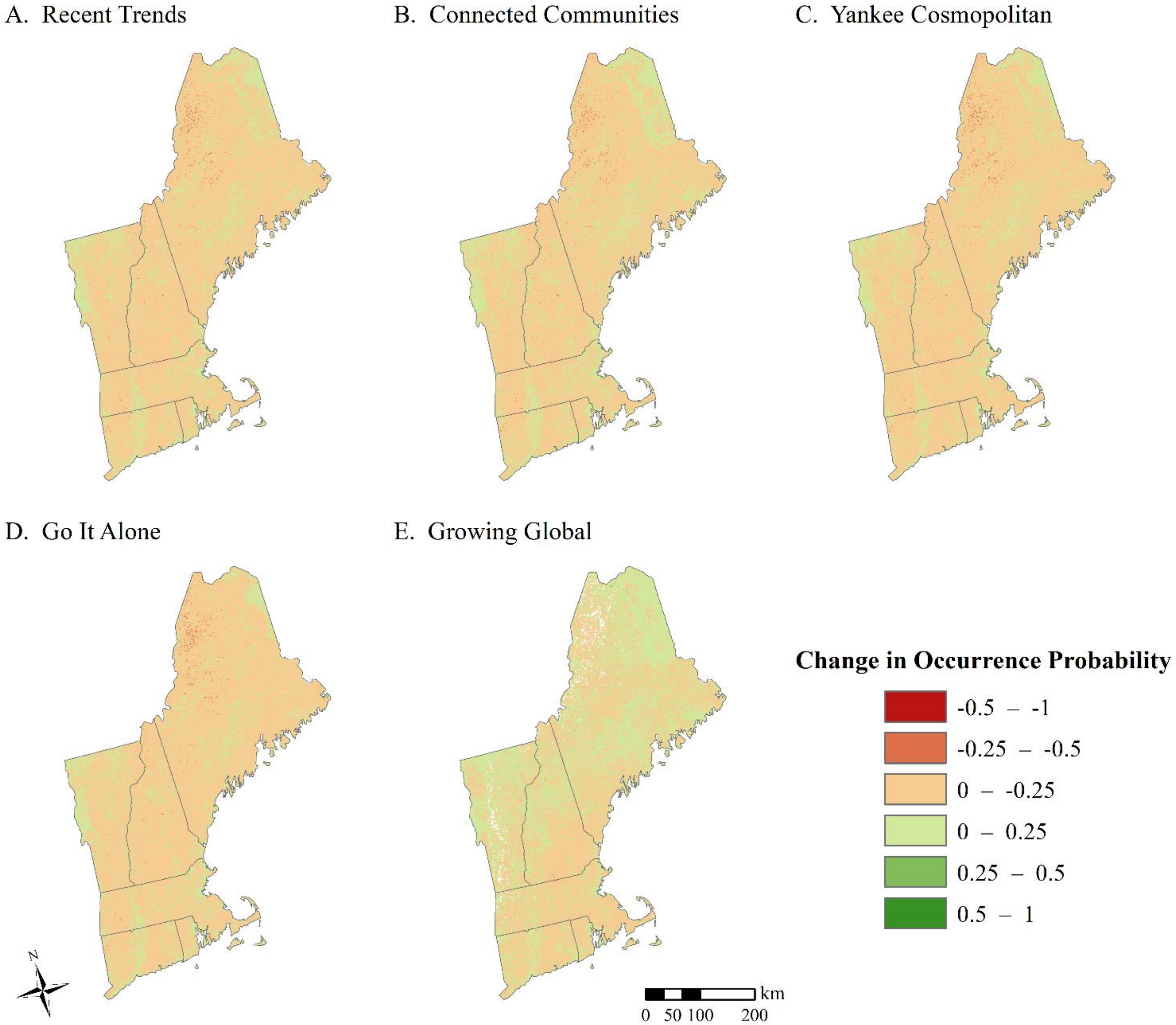

H. White-tailed deer

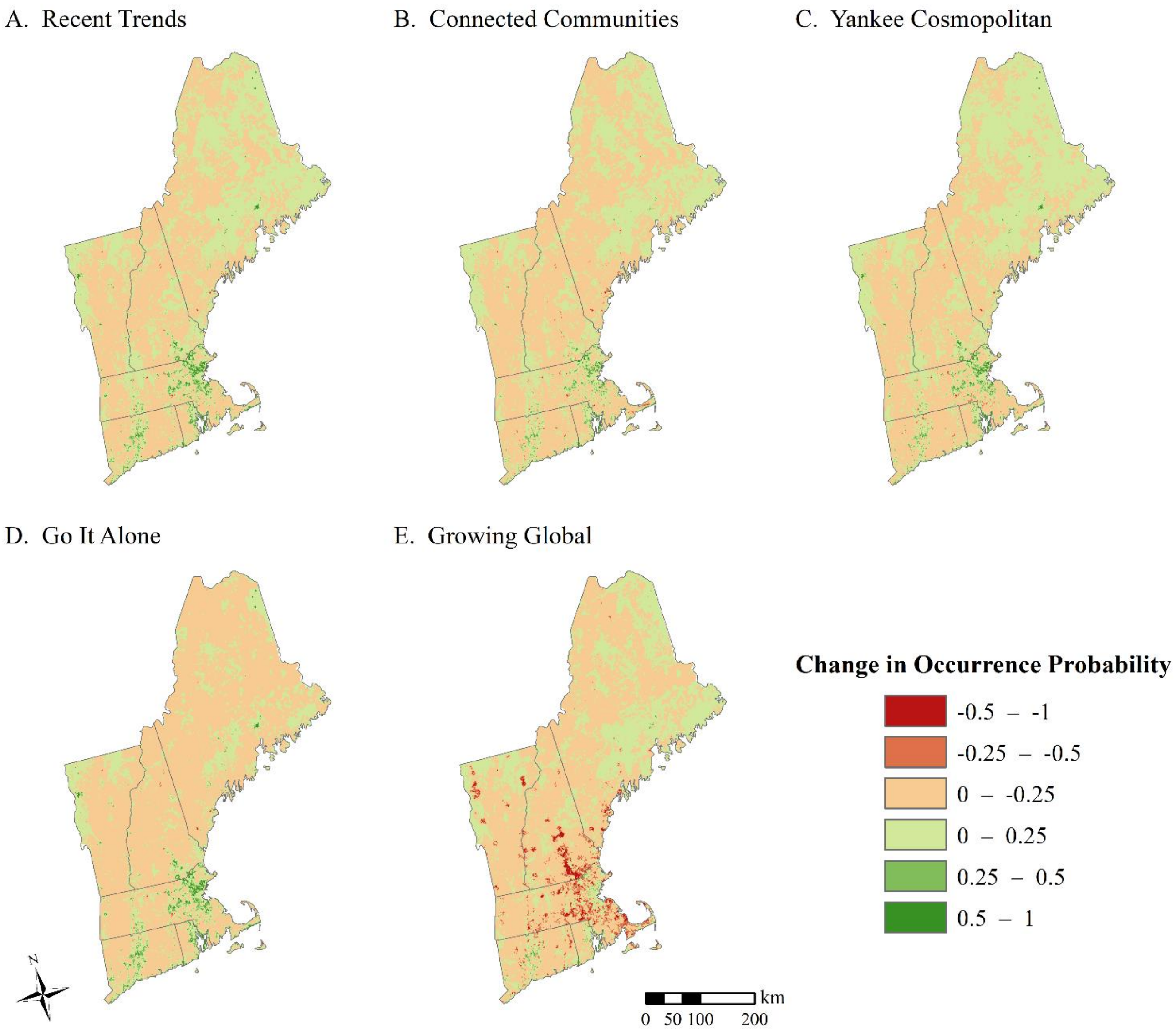

I. Wild turkey

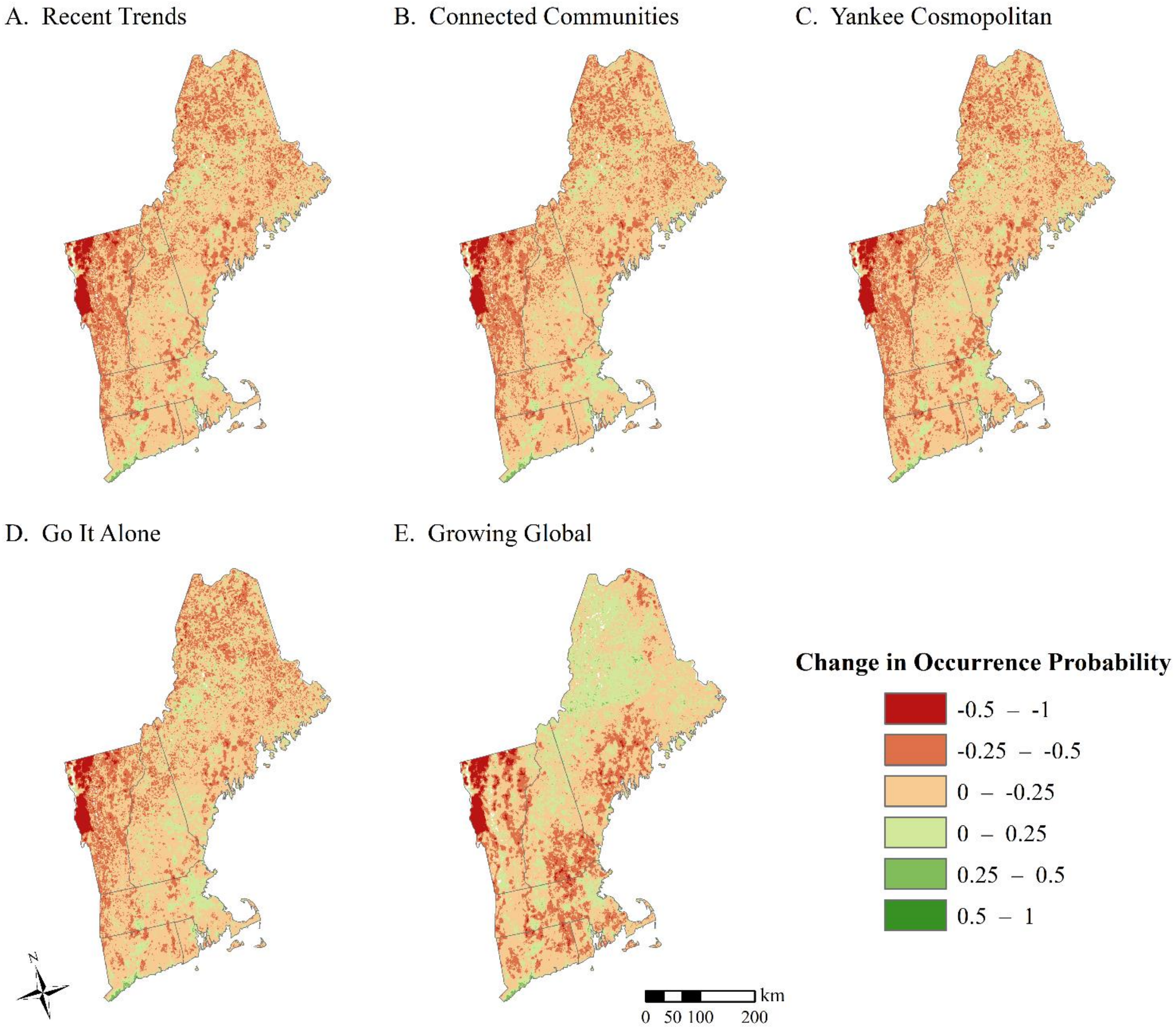

Supplement: S1 Fig — Distribution change was projected for nine wildlife species between current (2010) conditions and each of the NELFP scenarios: A) Business-As-Usual, B) Connected Communities, C) Yankee Cosmopolitan, D) Go It Alone, and E) Growing Global. Maps display changes in species probability of occurrence, derived from simulated distribution maps for 2010 and 2060 (see [34,62] for more details). (PDF) [file pone.0239525.s002.pdf]
