# Supplementary material for: Wildlife resistance and protection in a changing New England landscape
Source: PLoS One. 2020 Sep 24;15(9):e0239525. doi: 10.1371/journal.pone.0239525 (PMC7515594; doi:10.1371/journal.pone.0239525)
Supplement: S2 Fig — Map displays mean species resistance between 2010 and 2060 based on binary resistance maps for nine focal wildlife: American black bear, bobcat, coyote, moose, raccoon, red fox, striped skunk, white-tailed deer, and wild turkey. (PDF) [file pone.0239525.s003.pdf]

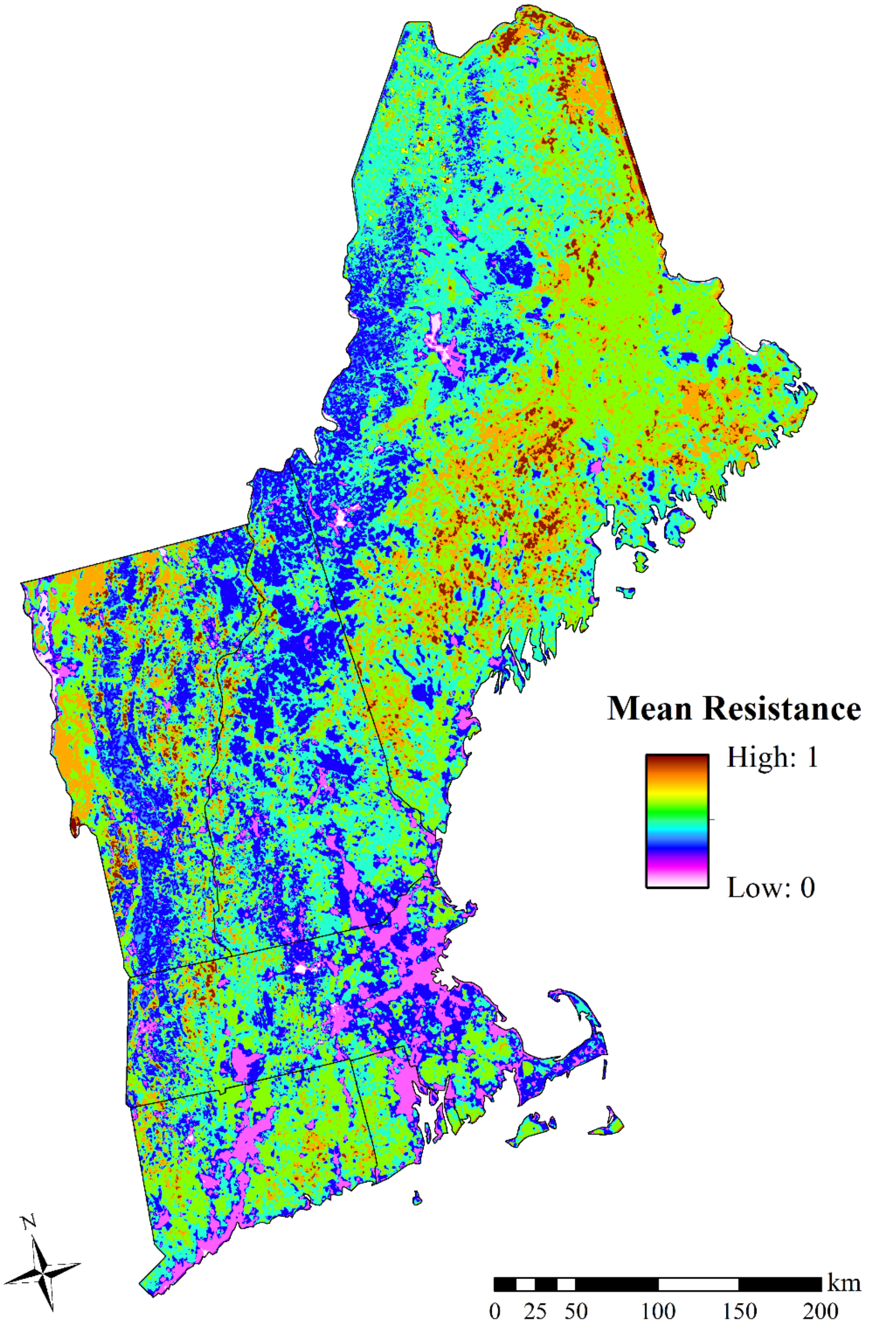

S2 Fig. Average focal species resistance throughout New England, USA.
Map displays mean species resistance between 2010 and 2060 based on binary resistance maps for nine focal wildlife: American black bear, bobcat, coyote, moose, raccoon, red fox, striped skunk, white-tailed deer, and wild turkey.
